# Genetic difference between two *Schistosoma japonicum* isolates with contrasting cercarial shedding patterns revealed by whole genome sequencing[Fn FN1]

**DOI:** 10.1051/parasite/2023061

**Published:** 2023-12-12

**Authors:** Hui-Ying Sun, Jie-Ying Zhang, Han-Xiang Zhang, Qing Xu, Da-Bing Lu

**Affiliations:** 1 Department of Epidemiology and Statistics, School of Public Health, Jiangsu Key Laboratory of Preventive and Translational Medicine for Geriatric Diseases, MOE Key Laboratory of Geriatric Diseases and Immunology, Suzhou Medical College of Soochow University 199 RenAi Road, Industrial Park Avenue Suzhou Jiangsu 215123 PR China

**Keywords:** *Schistosoma japonicum*, Whole genome sequencing, Genetic differentiation, Cercarial emergence pattern

## Abstract

*Schistosoma japonicum* is one of the major infectious agents of human schistosomiasis, mainly endemic in China and the Philippines. We have previously reported the finding of two schistosome isolates, each with a different cercarial emergence pattern adapted to their different hosts. However, there are currently no whole-genome sequencing studies to investigate the underlining genetics of the adaptive traits. We sampled schistosomes in 2013 and 2020 from a hilly area Shitai (ST) and a marshland area Hexian (HX) of Anhui, China. Ten to 15 male or female adult worms from each site/year were sent for whole genome sequencing. Genetics were analyzed, and selection signals along genomes were detected. Gene enrichment analysis was performed for the genome regions under selection. The results revealed considerable genetic differentiation between the two isolates. The genome “windows” affected by natural selection were fewer in ST (64 windows containing 78 genes) than in HX (318 windows containing 276 genes). Twelve significantly enriched genes were identified in ST, but none in HX. These genes were mainly related to specific DNA binding and intercellular signaling transduction. Some functional region changes identified along the genome of the hilly schistosome may be related to its unique late afternoon cercarial emergence.

## Abstract (fr)

*Schistosoma japonicum* est l’un des principaux agents infectieux de la schistosomiase humaine, principalement endémique en Chine et aux Philippines. Nous avons précédemment rapporté la découverte de deux isolats de schistosomes, chacun présentant un schéma différent d’émergence de cercaires, adapté à leurs différents hôtes. Cependant, il n’existe actuellement aucune étude de séquençage du génome entier pour comprendre la génétique sous-jacente aux traits adaptatifs. Nous avons échantillonné des schistosomes en 2013 et 2020 dans une zone de collines de Shitai (ST) et une zone marécageuse de Hexian (HX) de l’Anhui, en Chine. Dix à 15 vers adultes mâles ou femelles de chaque site/année ont été envoyés pour séquençage du génome entier. La génétique a été analysée et des signaux de sélection le long des génomes ont été détectés. Une analyse d’enrichissement génétique a été réalisée pour les régions du génome sélectionnées. Les résultats ont révélé une différenciation génétique considérable entre deux isolats. Les « fenêtres » du génome affectées par la sélection naturelle étaient moins nombreuses dans ST (64 fenêtres contenant 78 gènes) que dans HX (318 fenêtres contenant 276 gènes). Douze gènes significativement enrichis ont été identifiés dans ST mais aucun dans HX. Ces gènes étaient principalement liés à la liaison spécifique à l’ADN et à la transduction de la signalisation intercellulaire. Certains changements de régions fonctionnelles identifiés le long du génome du schistosome des collines peuvent être liés à son émergence cercarienne exceptionnelle en fin d’après-midi.

## Introduction

Schistosomiasis is a parasitic disease caused by trematode flukes of the genus *Schistosoma* and is estimated to affect at least 230 million people worldwide. *Schistosoma japonicum* (digenean trematode) is mainly endemic in China and partly in the Philippines [19, 52]. Schistosomiasis in humans in mainland China is caused by *S. japonicum* only [2]. After nearly 70 years of schistosomiasis control in China, great achievements have been obtained, with infections in both humans and livestock reduced to a much lower level across the country [16]. As a consequence, the central government of China has set the goal to eliminate this disease by 2030 [3]. However, many challenges remain, one of which may be attributable to its zoonotic transmission, as over 40 species of wild and domestic animals can serve as reservoir hosts for *S. japonicum* [19].

*Schistosoma japonicum* has a complex life cycle, involving an obligatory molluscan intermediate host of the genus *Oncomelania* and a mammalian definitive host. In fresh water, miracidia hatch from eggs, which are produced by sexual reproduction in definitive hosts, and actively penetrate a snail within which asexual reproduction occurs. The infected snail releases free-swimming cercariae that infect humans or reservoir hosts via skin penetration [6]. Snail-to-mammal transmission depends on temporal coincidence between the cercariae and the vertebrate because vertebrates are generally in contact with the water for a short length of time, and cercariae have a short lifespan. Cercarial infectivity declines rapidly after a few hours post-emergence from a snail, whereas their mortality increases [47]. Therefore, the chronobiological characteristics of cercarial emission naturally play a paramount role in schistosome transmission.

Cercarial emergence rhythms are regarded as adaptive activities of parasites to maximize the probability of encountering the right host [7, 28, 31, 41], and moreover, are probably genetically determined [35, 42]. Several studies have been carried out to show the intraspecific polymorphisms of cercarial emergence patterns [28, 33, 39, 41]. For example, on the island of Guadeloupe, *S. mansoni* cercariae with an early shedding pattern were observed in an urbanized area, where humans were highly infected; whereas cercariae with a late shedding pattern were observed in a sylvatic site, where rats were heavily infected [41]. In Anhui (China), *S. japonicum* cercariae from a hilly region displayed a late afternoon emergence pattern consistent with a nocturnal rodent reservoir, while those from a marshland region showed an early pattern compatible with a diurnal cattle reservoir [28, 39]. The variations of cercarial emergence between schistosome isolates were considered to be related to different final hosts. A number of studies, based on COX1 or microsatellites [28, 32, 36, 43], have attempted to elucidate any genetic differences between parasites with respect to their biological traits, definitive hosts, or possible habitat types. Evidence from morphological to molecular level indicated that *S. japonicum* in mainland China may comprise a strain complex, but the low- and middle-valley (including Anhui province) of the Yangtze River were endemic with the same *S. japonicum* strain [18]. Recent advances in whole genome sequencing technology (WGS) make it feasible to study the genetic architecture of schistosomes with different traits [17, 21], as genome-wide genetic variation can provide a more comprehensive and unbiased framework to identify genomic regions associated with the phenotypic traits.

We have previously reported that in Shitai (ST), a hilly/mountain area (HM) of Anhui, *S. japonicum* cercariae had a unique late afternoon emergence pattern, and the definitive host-parasite system was driven by local rodents in spite of high infections in humans [27, 28, 37]. Moreover, our recent research suggested that there was an increased prevalence of *S. japonicum* infections in rodents in HM regions across China [53]. We therefore hypothesized that the specific late-afternoon emergence pattern of *S. japonicum* was the result of ongoing selection posed by definitive host rodents. As there are no studies to explain the underlining genetics of this kind of adaptive traits with WGS, in this study we sequenced two *S. japonicum* isolates of Anhui, each with a different cercarial shedding pattern, and then analyzed their genetic relationships with the purpose of detecting any changes in nucleotide diversity and allele frequencies in response to the unique chronobiological characteristics.

## Materials and methods

### Material and sequencing

**Figure 1:**
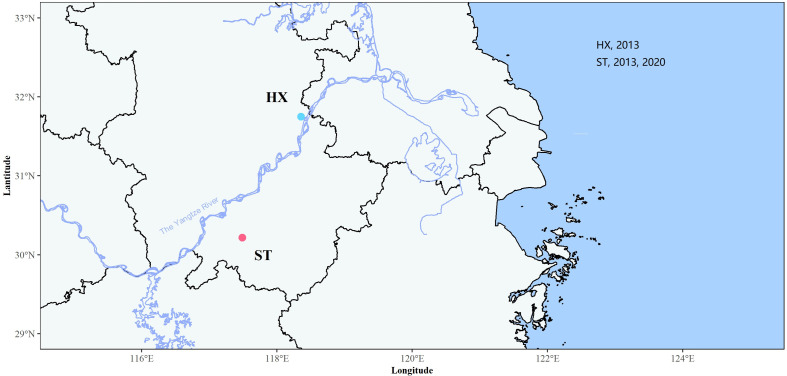
Map of geographical locations of research sites.

*Schistosoma japonicum* were sampled from two sites in Anhui province, China. One site was in Shitai county (ST), a hilly area where *S. japonicum* was observed with a late afternoon cercarial shedding pattern and rodents were the main reservoir; another was in Hexian (HX), a lake/marshland area (LM) where *S. japonicum* was observed with a diurnal shedding of cercariae emergence [28] and bovines were the main reservoir. Details on the field samples used in this study are given in Table 1 and Figure 1. Briefly, intermediate host *Oncomelania hupensis hupensis* snails [9] were captured via routine field surveys in 2013 and 2020, and isolation of infected snails was performed by releasing cercariae in the laboratory. Infected snails were identified in Shitai in 2013 and 2020 and in Hexian in 2013 only. Cercariae released from infected snails of each site/year were separately and subsequently used to establish their corresponding *S. japonicum* isolates in mice, in which mice were individually exposed to cercariae from a single infected snail. Four weeks later, adult worms were perfused from infected mice and frozen in absolute ethanol, as a future genetic resource. The Ethics Committee of Soochow University reviewed and approved this research protocol (No. 81971957). Ten to 15 male or female adult worms, either batch of which derived from single infected snails, were selected from each site/year (*i.e.*, ST2020m and ST2020f, for males and females from ST in 2020, respectively; ST2013m and ST2013f, for males and females from ST in 2013, respectively; HX2013m and HX2013f, for males and females from HX in 2013, respectively), and were then sent to Novogene (Tianjin, China) for whole genome sequencing. Pooled DNA was extracted using the DNeasy Blood & Tissue Kit (QIAGEN, Hilden, Germany), according to the manufacturer’s instructions. Paired-end reads (2 × 150 bp) for each sample were produced on a NovaSeq 6000 system (Illumina, San Diego, CA, USA). The data that support the findings of this study are openly available in the BioProject database at https://dataview.ncbi.nlm.nih.gov/, reference No. PRJNA793934.

**Table 1:** Geographical information of *S. japonicum* samples.

Sample code	Locality	Ecological setting	Collection year	Schistosome
ST2020m	Shitai, Anhui	HM	2020	Male
ST2020f	Shitai, Anhui	HM	2020	Female
ST2013m	Shitai, Anhui	HM	2013	Male
ST2013f	Shitai, Anhui	HM	2013	Female
HX2013m	Hexian, Anhui	LM	2013	Male
HX2013f	Hexian, Anhui	LM	2013	Female

Note: HM, Hilly and mountainous areas; LM, Lake and marshland areas.

### Read mapping and variant calling

Raw reads were filtered with FASTP [4] with the parameters -q 5 -u 50 -n 15, and aligned to the reference genome (ASM636876v1) [29] with BWA-MEM (version 0.7.17) [24] with default parameters. The aligned bam files were then sorted using SAMtools [25], and PCR duplicate reads were marked with Picard-MarkDuplicates (http://broadinstitute.github.io/picard/). Mapping statistics were estimated with SAMtools. The Genome Analysis Toolkit (GATK) [10, 30] was used for variant calling. By running HaplotypeCaller, genomic variant call format (GVCF) files were generated for each sample. These GVCF files were next combined into a single GVCF file, which was then used to identify single nucleotide polymorphisms (SNPs) and small indels with GenotypeGVCFs. SNPs and small indels were further filtered using the following criteria: (1) SNPs were filtered with QD < 2.0, QUAL < 30.0, MQ < 40.0, FS > 45.0, SOR > 3.0, MQRankSum < −12.5, and ReadPosRankSum < −8.0, and indels with QD < 2.0, QUAL < 30.0, MQ < 40.0, FS > 200.0 and SOR > 10.0, and ReadPosRankSum < −20.0; (2) only variants with two alleles were retained and missing data prohibited by using VCFtools (version 0.1.13) [8]; (3) linkage disequilibrium pruning was performed with PLINK (version 1.9) (https://zzz.bwh.harvard.edu/plink/ld.shtml) using a 1-kb window with a step size of 10 SNPs and an *r* ^2^ threshold of 0.1; (4) variants with a minor allele frequency (MAF) of <0.05 were removed. All identified variants were annotated with Snpeff (version 5.0e) [5]. The VCF files including samples of each site/year were obtained by using VCFtools (version 0.1.13), and annotated with Snpeff (version 5.0e). SNPs and indels were categorized based on their positions on the genome (including intergenic regions, exons, introns, splicing sites, untranslated regions, transcripts, and 1-kb upstream and downstream regions) and on their effects (*i.e.*, missense, nonsense, and silent mutations). Singleton SNPs in each sample were also calculated with VCFtools.

### Genetic differentiation

Principal component analysis (PCA) was performed on the filtered SNP sets with PLINK [38]. For each site/year, nucleotide diversity (*π*) was calculated within a 100-kb window sliding in 10-kb steps, and Tajima’s *D* was calculated within a nonoverlapping 100-kb window with VCFtools [22]. Pairwise genetic differentiation (*F*_ST_ per gene between sampling sites/years) was calculated within a 100-kb window sliding in 10-kb steps using VCFtools [22].

### Identification of selected regions in genomes

We conducted tests to detect selection signals along the genomes of ST and HX. The *π* ratios (*π*_ST_/*π*_HX_) and *F*_ST_ values were calculated by the sliding-window approach (a 100-kb window sliding in a 10-kb step). To avoid spurious selection signals, windows containing fewer than 10 informative sites from both *π* and *F*_ST_ analyses were discarded. We used an empirical procedure [26] and selected the windows simultaneously with significantly low or high *π* ratios (the 5% left or right tails of the distribution) and significantly high *F*_ST_ values (the 5% right tail of the distribution). These windows were regarded as the regions with strong selective sweep signals, which should harbor candidate genes that undergo a selective sweep. Candidate genes of selective regions were identified using annotations of the reference [29] with Snpeff (version 5.0e) [5]. In order to understand what functions these genes were involved in, we tested for enrichment of gene ontology (GO) terms by comparing to the reference genome with the clusterProfiler package [49]. The Benjamini-Hochberg correction was applied and significantly enriched GO terms were identified at a corrected *p-*value of <0.05.

### Impact of sampling time on selection

As ST included two different years (i.e., 2013 and 2020) of samples, we further analyzed allele frequency changes for every SNP on the comparisons of ST2013 *vs.* ST2020 and HX2013 *vs.* ST2013 by performing Fisher’s exact tests (FET) and Cochran-Mantel-Haenszel tests (CMH) with popoolation2 [22]. For FET test, the six sorted and marked BAM files were merged into three groups according to sampling site/year using SAMtools [25]. For CMH test, the six files were kept separate and formed pairs as following: 2 × (ST2013 *vs.* ST2020) and 2 × (HX2013 *vs.* ST2013). Pile-up files were generated with SAMtools’ mpileup (-d 500 –min-MQ 30 –min-BQ 30 –adjust-MQ 50) using the BAM files as input. The pile-up files were converted into synchronized files (popoolation2 mpileup2sync.pl –min-qual 20), and sequences around indels +5 bp were identified (popoolation2 identify-indel-regions.pl –min-count 2 –indel-window 5) and removed (popoolation2 filter-sync-by-gtf.pl). Significance with a genome-wide Bonferroni’s correction (0.05/(number of genome-wide SNPs)) was used in both tests. Pairwise genetic differentiation (*F*_ST_ per gene) was calculated using popoolation2 (popoolation2 fst-sliding.pl –pool-size 1000 –min-count 4 –min-coverage 20 –max-coverage 2% –window-size 1000000 –step-size 1000000) [22], with the annotated synchronized file (popoolation2 create-genewise-sync.pl) as an input (popoolation2 create-gene-wise-sync.pl).

## Results

### Sequencing and mapping

Six schistosome samples (*i.e.*, ST2013m, ST2013f, ST2020m, ST2020f, HX2013m, and HX2013f) generated a total of 84.49 Gb of raw sequencing data. After filtering sequences of low quality, 0.55 billion reads were left with an average mapping depth of 35× on the reference genome. The mapping rate was between 95.70% and 96.27%, and the median coverage ranged from 93.58% to 95.18% (Table 2).

**Table 2:** Summary of mapping statistics.

Sample code	Sequencing data (Gb)	Total reads	Reads aligned	Mapping rate %	Mismatch rate	Average quality	Reads aligned in pairs	Reads aligned in pairs %	Mean read length	Average insert size	Median genome coverage %	Depth
ST2020m	13.77	90,489,190	87,048,798	96.20%	0.03	35.8	86,444,586	99.31%	150	340.6	93.58%	34
ST2020f	13.98	91,349,560	87,786,731	96.10%	0.03	35.8	87,209,346	99.34%	150	334.3	93.85%	35
ST2013m	14.40	94,724,430	90,947,858	96.01%	0.03	35.8	90,335,934	99.33%	150	330.7	93.61%	36
ST2013f	14.01	91,737,504	87,797,151	95.70%	0.03	35.8	87,234,312	99.36%	150	318.1	93.69%	35
HX2013m	14.21	93,295,110	89,812,077	96.27%	0.02	35.8	89,265,388	99.39%	150	326.2	95.04%	36
HX2013f	14.12	92,149,586	88,666,850	96.22%	0.02	35.8	88,125,512	99.39%	150	332.7	95.18%	35

### Variant identification

Single nucleotide variants varied among samples, mostly between two schistosome isolates. Heterozygous SNPs ranged from the lowest 2,093,493 in ST2013m to the highest 4,335,470 in HX2013m, while homozygous SNPs were in range of the lowest 727,770 in HX2013m to the highest 4,335,470 in ST2013m. The Ts/Tv (transitions/transversions) varied between 1.807 in ST2013f and ST2020m and 1.837 in HX2013f. The average Ts/Tv (transitions/transversions) of variation was 1.82. Singleton SNPs ranged from the lowest 196,368 in ST2013m to the highest 445,959 in HX2013f. Nonsynonymous SNPs varied between 39982 in HX2013m and 81449 in ST2020f (see Table 3).

**Table 3:** Statistics of SNPs of six samples.

Sample	HX2013f	HX2013m	ST2013f	ST2013m	ST2020f	ST2020m
SNP	5063240	4575574	6207199	6063206	6216959	6193470
Heterozygous	4335470	3799796	2370989	2093493	2380273	2313843
Homozygous	727770	775778	3836210	3969713	3836686	3879627
Transitions	3748405	3465319	6465041	6459947	6473971	6484924
Transversions	2042605	1886033	3578368	3572972	3579674	3588173
Ts/Tv	1.835	1.837	1.807	1.808	1.809	1.807
Singleton SNPs	415678	445959	217500	196368	220987	228412
Doubleton SNPs	15430	13938	17908	27213	19596	18133
Nonsynonymous	47596	39982	80858	77893	81449	78683
Synonymous	49874	41546	88844	85980	89251	87640

### Genetic differentiation

**Figure 2:**
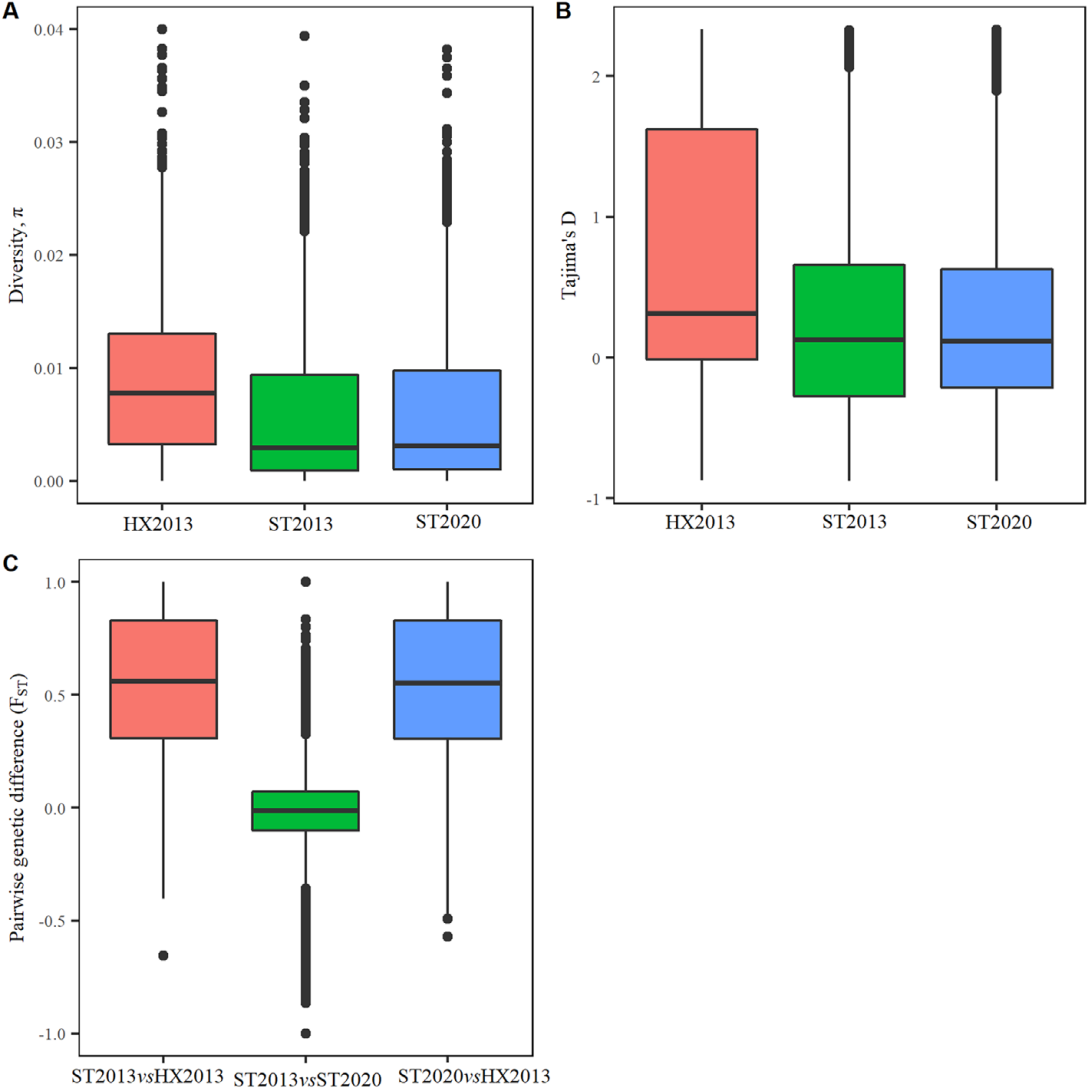
Box plots of diversity, Tajima’s D of *S. japonicum* and their *F*_ST_. (A) Nucleotide diversity estimated in 100-kb windows sliding in 10-kb steps throughout the genome. (B) Tajima’s D estimated within a nonoverlapping 100-kb window throughout the genome. (C) pairwise *F*_ST_ computed in 100-kb windows sliding in 10-kb steps throughout the genome.

**Figure 3:**
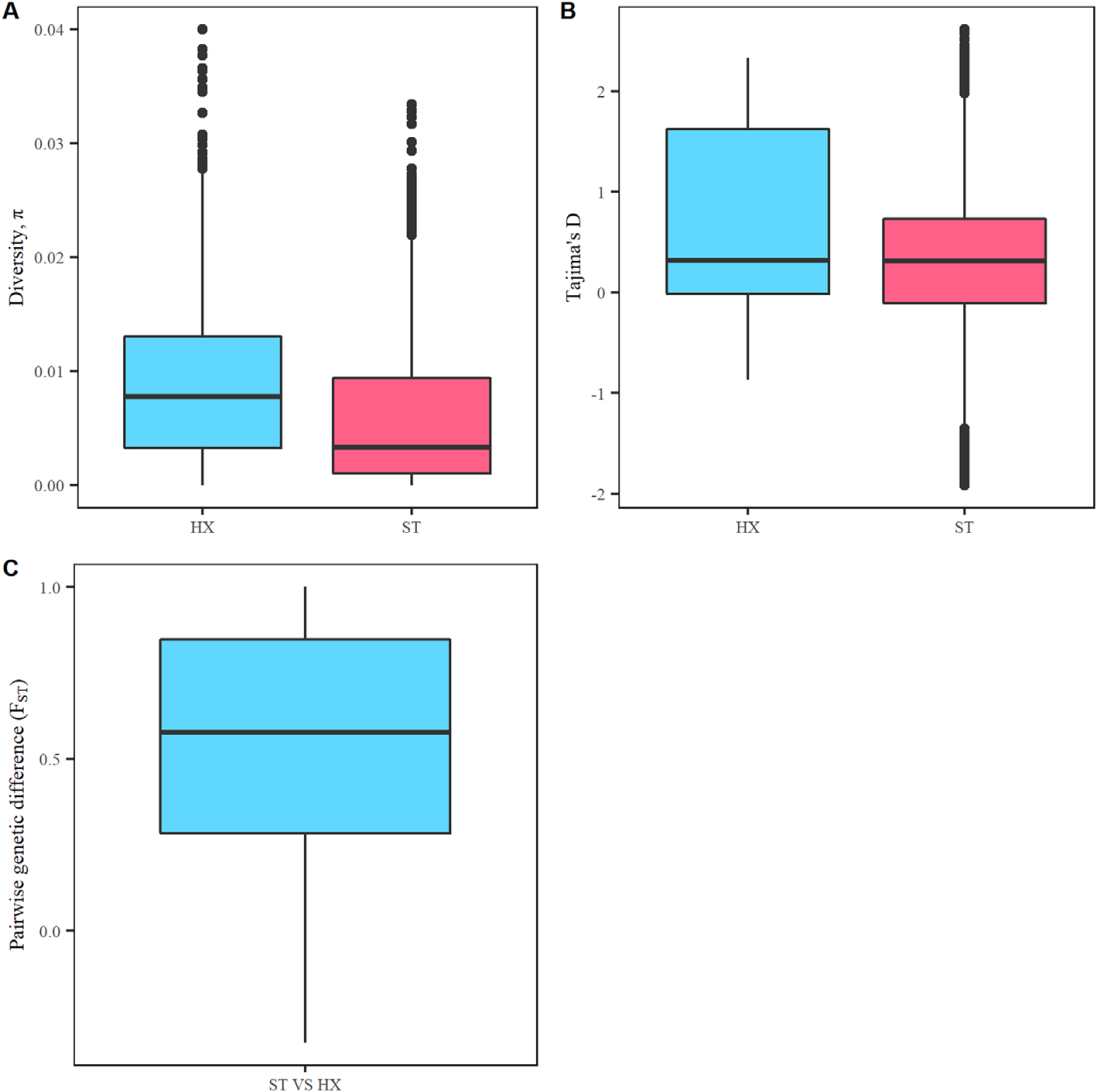
Box plots of diversity and relationship of two sample groups. (A) Nucleotide diversity estimated in 100-kb windows sliding in 10-kb steps throughout the genome. (B) Tajima’s D estimated within a nonoverlapping 100-kb window throughout the genome. (C) *F*_ST_ between computed in 100-kb windows sliding in 10-kb steps throughout the genome.

PCA analysis classified schistosome samples according to their sampling site and year, in which the percentage of the variation in genetic distances explained by each PC is 46.9 and 34.9% for PC1 and PC2, respectively. When samples were grouped based on sampling site and year (*i.e.*, ST2013m and ST2013f into ST2013, ST2020m and ST2020f into ST2020, and HX2013m and HX2013f into HX2013), the pairwise genetic differentiation (*F*_ST_) between HX2013 and ST2013 or ST2020 were 0.55 and 0.54, respectively, showing a great genetic difference; whereas the index between ST2013 and ST2020 was −0.01, indicating almost no difference between both. The values of nucleotide diversity (*π*) of ST2013 and ST2020 was almost the same, and so for the Tajima’s *D*. Moreover, both *π* and Tajima’s *D* were much lower when compared to HX2013 (see Table 4 and Fig. 2). We then further grouped samples based on sampling sites, namely ST and HX. The lower nucleotide diversity (*π*) was in ST (5.61 × 10^−3^) and higher in HX (8.48 × 10^−3^) (Fig. 3A). Selective neutrality, tested with Tajima’s *D*, showed that ST had more windows observed at extremely high or low values. The overall value of Tajima’s *D* computed along genomes in ST (0.29) was lower than that in HX (0.67) (Fig. 3B). Significant differences for *π* and Tajima’s *D* value distributions between both sites were observed (*p*-value < 0.05; two-sample Kolmogorov-Smirnov test). *F*_ST_ revealed a high degree of genetic differentiation between two sites (mean genome-wide *F*_ST_ = 0.56) (Fig. 3C).

**Table 4:** *π* and *F*_ST_ of three groups.

Sample group	Number of SNPs	*π* (×10^−3^)	*F*_ST_ with ST2013	*F*_ST_ with ST2020
ST2013	36868861	5.57	–	−0.01
ST2020	37882880	5.73	−0.01	–
HX2013	54244144	8.48	0.55	0.54

### Genome-wide selective sweep analysis

**Figure 4:**
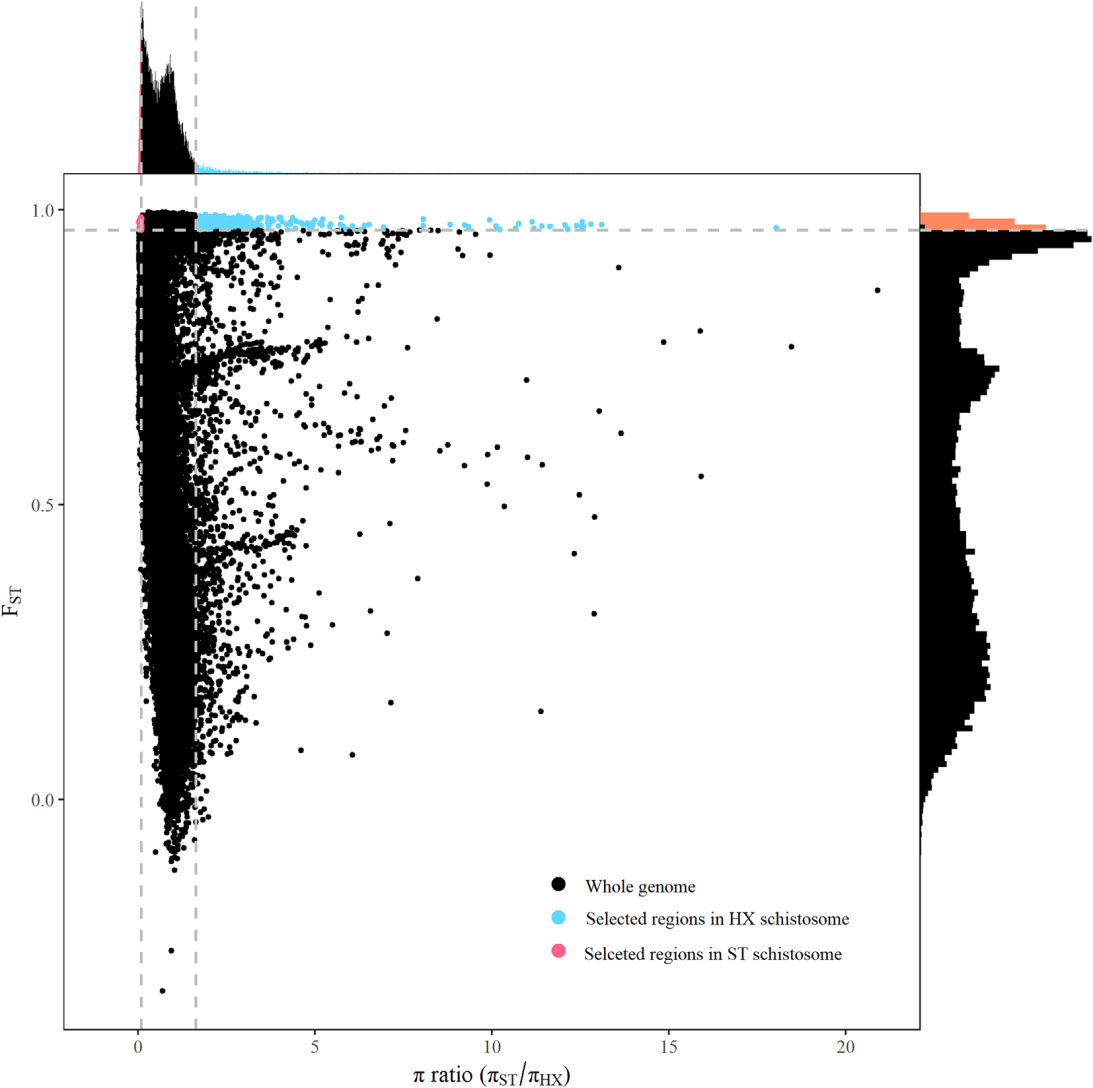
Distribution of *π* ratios (*π*_ST_/*π*_HX_) and *F*_ST_ values calculated in 100-kb windows sliding in 10-kb steps throughout the genome. Data points colored red and blue were identified as selected regions in ST (red dots) and in HX (blue dots), respectively. These points correspond to the 5% left and right tails of the empirical *π* ratio distribution, where the *π* ratios are 0.092 and 1.643, respectively (vertical dashed lines), and the 5% right tail of the empirical *F*_ST_ distribution, where *F*_ST_ is 0.965 (horizontal dashed line).

**Figure 5:**
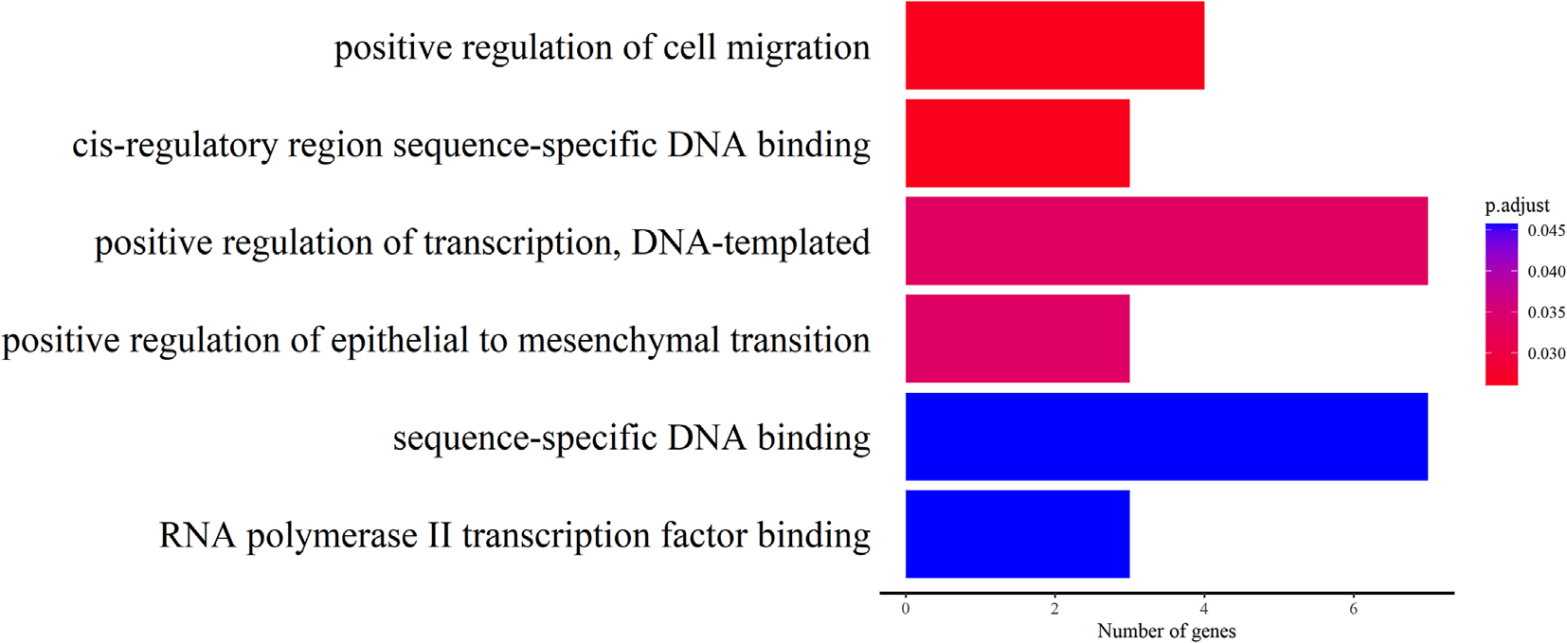
Gene ontology enrichment analysis of genes from genome regions with strong selective signals.

A sliding-window approach was applied to identify selection signals on genomes. Out of 36,389 windows examined 424 were discarded as they contained fewer than 10 informative sites from both *π* value and *F*_ST_ analysis. Strong selective sweep signals were detected in the remaining windows. The genome regions in ST affected by natural selection were fewer than in HX. In ST, strong signals were detected within 64 windows containing 78 genes; whereas in HX within 318 windows containing 276 genes (Fig. 4; Supplemental Tables S1 and S2). ST had lower levels of polymorphism than HX (median *π*_ST_/*π*_HX_ = 0.658), and selection regions in each site showed higher levels of selection statistics (*F*_ST_ and Tajima’s *D*) (Supplemental Fig. S1). The results suggested that selection may exist in shaping the genome, resulting in changes in phenotypic and/or behavioral traits of schistosomes. However, no significantly enriched genes were identified in the selection regions in HX, but in ST about 12 significantly enriched genes were identified (Supplemental Table S3). The functions of those genes involve positive regulation of cell migration, cis-regulatory region sequence-specific DNA binding, positive regulation of transcription, DNA-templated, positive regulation of epithelial to mesenchymal transition, sequence-specific DNA binding and RNA polymerase Ⅱ transcription factor binding (Fig. 5). These enriched genes showed a high level of genetic differentiation (average *F*_ST_ = 0.91) and were mainly related to specific DNA binding and intercellular signaling transduction (Table 5).

**Table 5:** Information of enriched genes of *S. japonicum* from ST.

Gene ID	Scaffold	Tajima’s *D*	*F*_ST_	Protein ID	Product
EWB00_001158	SKCS01000144.1	−0.09	0.79	TNN15636.1	Disintegrin and metalloproteinase domain-containing protein
EWB00_001161	SKCS01000144.1	−1.05	0.84	TNN15643.1	Homeobox protein
EWB00_002585	SKCS01000182.1	0.06	0.97	TNN13876.1; TNN13877.1; TNN13878.1; TNN13879.1	Remodeling and spacing factor 1 isoform 2; Remodeling and spacing factor 1 isoform 1
EWB00_003259	SKCS01000202.1	−0.28	0.90	TNN13009.1; TNN13010.1	Syntenin-1 isoform 1; Syntenin-1 isoform 2
EWB00_004024	SKCS01000026.1	0.44	0.94	TNN20442.1; TNN20443.1; TNN20444.1; TNN20445.1; TNN20446.1; TNN20447.1	Suppressor of hairless protein isoform 1; Suppressor of hairless protein isoform 2; Suppressor of hairless protein isoform 3; Suppressor of hairless protein isoform 4; Suppressor of hairless protein isoform 5
EWB00_008464	SKCS01000470.1	−0.51	0.92	TNN06290.1; TNN06291.1; TNN06292.1; TNN06293.1	GATA-binding factor A isoform 1; GATA-binding factor A isoform 2; GATA-binding factor A isoform 3
EWB00_008467	SKCS01000470.1	–	0.94	TNN06298.1	Zinc finger protein
EWB00_010839	SKCS01000087.1	0.21	0.93	TNN17783.1	Transcriptional enhancer factor TEF-1
EWB00_010840	SKCS01000087.1	−1.31	0.95	TNN17784.1	Transcriptional enhancer factor TEF-1
EWB00_010970	SKCS01000090.1	0.86	0.93	TNN17525.1	Histone-lysine N-methyltransferase NSD2
EWB00_010973	SKCS01000090.1	0.40	0.95	TNN17529.1	Paired box protein
EWB00_010978	SKCS01000090.1	0.89	0.90	TNN17535.1; TNN17536.1	Mothers against decapentaplegic member 3 isoform 1; Mothers against decapentaplegic member 3 isoform 2

### Impact of sampling time on selection

To investigate whether the two different years of ST samples affect the above selective analysis, we further performed both FET and CMH tests to examine SNP differences on comparisons of ST2013 *vs*. HX2013 and ST2013 *vs*. ST2020 (Supplemental Fig. S2). For HX2013 *vs*. ST2013, FET yielded roughly 2.36 million SNPs in two merged groups, whereas CMH test contained 0.37 million SNPs throughout the comparisons. A high degree of concordance between FET and CMH (Pearson’s correlation; *r* = 0.98) was identified when comparing the SNPs shared between the two tests (*n* = 0.36 million). As for ST2013 *vs*. ST2020, about 1.36 million SNPs were used in FET and 0.92 million in CMH test. Correlation analysis between FET and CMH tests revealed a high level of concordance (*n* = 0.19 million; Pearson’s correlation; *r* = 0.96). Both FET and CMH tests between HX2013 and ST2013 included higher number of significant SNPs than between ST2013 and ST2020. Moreover, the SNPs of the enriched genes (red dots in Supplemental Fig. S2) showed differences between HX2013 and ST2013, but none between ST2013 and ST2020. Except for the SNPs of five genes (EWB00_003259, EWB00_008464, EWB00_008467, EWB00_010839, and EWB00_010840) which showed significant changes above the genome-wide correction in the FET but not in the CMH test, the SNPs of all previously found selective genes showed significant changes in both FET and CMH tests. The pairwise *F*_ST_ of these genes showed a high level of difference between ST2013 and HX2013, while none between ST2013 and ST2020 (Supplemental Fig. S3).

## Discussion

With the available genome maps of *S. haematobium* [23], *S. mansoni* [24], and *S. japonicum* [25, 26], whole genome sequencing was used to reveal the population history of *S. mansoni* [27], to investigate introgression events of *S. bovis* and *S. haematobium* [28], and to evaluate the village-level relatedness and genetic diversity of *S. japonicum* [29]. In this work, using whole-genome pooled sequencing (pool-seq), we sequenced and analyzed genomes of six *S. japonicum* samples from two sites, each endemic for *S. japonicum* with a different cercarial emergence pattern. More than 11.87 million SNPs were identified, and two *S. japonicum* isolates showed great genetic differentiation and contrasting diversities. Although the genome region “windows” affected by natural selection were fewer in ST than in HX, more involved genes were identified in ST. The selective regions and relative genes on the schistosome genome might be related to its unique chronobiological characteristics of cercarial emergence.

In this study, the results from whole genome sequences of *S. japonicum* showed significant genetic differentiation between two isolates. Rudge *et al*. [36] once reported strong genetic differentiation in *S. japonicum* between two schistosome isolates and suggested that this differentiation may be associated with the contrasting host reservoirs. Indeed, our previous work in Anhui identified that rodents may be the key host for maintaining *S. japonicum* transmission within HM regions, while bovines are key hosts only within LM regions [27, 28, 37]. As cercarial emergence is a heritable trait, shaped by the behavior of definitive hosts [7, 41, 42], in the view of evolution, *S. japonicum* cercariae from the HM region with a late afternoon emergence pattern may be the result of adaptation to nocturnal rodent reservoirs, while those from the LM with an early pattern may be the result of adaptation to diurnal cattle reservoirs [28, 39]. The genetic differentiation observed between the two schistosome isolates in this work may possibly underline their different biological traits, caused by definitive hosts.

Zhao *et al*. [51] demonstrated that, based on mitochondrial DNA, a much larger degree of genetic variation was observed in low-lying LM regions along the middle and lower reaches of the Yangtze River than in the HM regions in the upper reaches of the River. Similarly, in our work, we observed higher genetic diversity in HX than in ST. The greater genetic diversity and higher positive Tajima’s *D* in HX could result from balancing selection for the parasites, for example at the stage of asexual reproduction within intermediate host snails, as in the marshland annual floods may have brought about the widespread mixing and dispersal of snails across large geographical areas [12, 48]. However, the positive values of Tajima’s D in both HX and ST indicate that some regions in the genomes of the two *S. japonicum* isolates may be under selective pressure by definitive hosts or other factors.

More selective signals were detected on schistosome genomes from HX than from ST, suggesting that HX might receive more selective pressure. This may be explained by the fact that bovines and humans are the key hosts in LM regions [37], and as a major source of infection, have been given high priority for control interventions [45]; whereas rodents are the key hosts in HM regions and are not able to be targeted due to logistic difficulties [23]. Significantly enriched genes were identified in selection regions of ST only, suggesting that they might be related to the fact that the schistosomes may have long resided and been transmitted within local rodents. The number of enriched genes were up to 12 and their products are mainly related to transcription and intercellular signal transduction [11, 13, 15, 40, 44], such as zinc finger protein [11], transcriptional enhancer factor, mothers against decapentaplegic member 3 isoform 1, and mothers against decapentaplegic member 3 isoform 2 (SMAD) [40, 46]. The signaling pathways involving SMAD have components sharing high identity with mammalian orthologues. High expression of SMAD may, as identified in the liver of infected mice [50], imply that schistosomes can, in addition to utilizing their own pathways, exploit host growth factors as developmental signals [40]. We thus proposed that schistosome from ST may strengthen its transcription activities to increase the opportunity to invade the right host and prepare to exploit key signaling pathways of their host for growth and metabolism. We further found that one of these enriched genes is responsible for the product of histone-lysine N-methyltransferase. This is of interest as histone modifying enzymes have been selected as targets for druggable epigenetic targets in early-stage schistosome drug discovery projects [34]. In addition, the involved products also included suppressor of hairless protein (transcription factor partner of Notch receptor), which plays a possible role in Notch signaling in sensory architecture [44]. Regarding sensory structures, Hoffmann *et al.* [20] found that some components of the light detection system, for example *S. mansoni* rhodopsin (SmRHO), were consistent with the responsiveness of cercariae to light. Their findings were reminiscent of cercarial emergence, a biological trait controlled to some extent by illumination [1, 14]. More studies would be needed to explore the role of Notch signaling in sensory architecture on *S. japonicum* in the hilly area.

In conclusion, we found that the hilly/mountainous *S. japonicum* isolate with late afternoon emergence was genetically different from the LM isolate with a morning shedding pattern, although both sites are within the same province and with *O. h. hupensis* as intermediate hosts. Moreover, we identified certain functional region changes along the genome of the hilly isolate that might be related to the unique late shedding pattern of schistosome cercariae.

## Supplementary materials

The supplementary material of this article is available at https://www.parasite-journal.org/10.1051/parasite/2023061/olm. *Table S1*: Genes of select regions in *S. japonicum* from ST. *Table S2*: Genes of select regions in *S. japonicum* from HX. *Table S3*: GO enrichment results in *S. japonicum* from ST. *Figure S1*: Box plots of (A) *π* ratio (*π*_ST_/*π*_HX_), (B) *F*_ST_, and (C) Tajima’s *D* of selection regions throughout genomes. *Figure S2*: Fisher’s exact test (A, B) and CMH test (C, D) were employed to investigate any significant SNP frequency changes (−log10[p-value]) between the two groups. −log10(P) values for common SNPs obtained through performing FET and CMH tests were plotted together (on separate axes) in order to identify significant allele frequency changes of variants in both tests (E, F). Tests between HX2013 and ST2013 are shown in (A, C, and E), while tests between ST2013 and ST2020 are shown in (B, D, and F). The red lines represent genome-wide Bonferroni’s correction of *p-*value. Dots in red represent SNPs of enriched genes above the genome-wide Bonferroni’s correction level. *Figure S3*: *F*_ST_ values for entire genes calculated between HX2013 and ST2013 (A), between ST2013 and ST2020 (B), and between ST and HX (C). Dots in red represent SNPs of enriched genes.
