# Supplementary material for: Genetic difference between two Schistosoma japonicum isolates with contrasting cercarial shedding patterns revealed by whole genome sequencing
Source: Parasite. 2023 Dec 12;30:59. doi: 10.1051/parasite/2023061 (PMC10714679; doi:10.1051/parasite/2023061)
Supplement: Supplementary file 1 — Table S1: Genes of select regions in S. japonicum from ST. Table S2: Genes of select regions in S. japonicum from HX. Table S3: GO enrichment results in S. japonicum from ST. Figure S1: Box plots of (A) π ratio (πST/πHX), (B) FST, and (C) Tajima’s D of selection regions throughout genomes. Figure S2: Fisher’s exact test (A, B) and CMH test (C, D) were employed to investigate any significant SNP frequency changes (−log10[p-value]) between the two groups. −log10(P) values for common SNPs obtained through performing FET and CMH tests were plotted together (on separate axes) in order to identify significant allele frequency changes of variants in both tests (E, F). Tests between HX2013 and ST2013 are shown in (A, C, and E), while tests between ST2013 and ST2020 are shown in (B, D, and F). The red lines represent genome-wide Bonferroni’s correction of p-value. Dots in red represent SNPs of enriched genes above the genome-wide Bonferroni’s correction level. Figure S3: FST values for entire genes calculated between HX2013 and ST2013 (A), between ST2013 and ST2020 (B), and between ST and HX (C). Dots in red represent SNPs of enriched genes. [file parasite-30-59-s1.zip › Table S1.docx]

Table S1. Genes of selection regions in *S. japonicum* from ST.

| Gene_Id | Protein_id | Product |
| --- | --- | --- |
| EWB00_001158 | TNN15636.1 | Disintegrin and metalloproteinase domain-containing protein |
| EWB00_001159 | TNN15637.1;TNN15638.1;TNN15639.1;TNN15640.1 | Protein madd-4 isoform 1;Protein madd-4 isoform 2 |
| EWB00_001160 | TNN15642.1 | CCA tRNA nucleotidyltransferase 1 isoform 1 |
| EWB00_001161 | TNN15643.1 | Homeobox protein |
| EWB00_001162 | TNN15644.1 | Homeobox HOX3 |
| EWB00_001163 | TNN15645.1;TNN15646.1;TNN15647.1;TNN15648.1;TNN15649.1; | DnaJ subfamily B member 9 isoform 2,DnaJ subfamily B member 9 isoform 1 |
| EWB00_001164 | TNN15650.1 | hypothetical protein |
| EWB00_002582 | TNN13871.1 | Splicing factor 45 |
| EWB00_002583 | TNN13873.1;TNN13874.1;TNN13872.1; | hypothetical protein;hypothetical protein;hypothetical protein; |
| EWB00_002584 | TNN13875.1; | TRAF3-interacting protein |
| EWB00_002585 | TNN13876.1;TNN13877.1;TNN13878.1;TNN13879.1; | Remodeling and spacing factor 1 isoform 2;Remodeling and spacing factor 1 isoform 1 |
| EWB00_002586 | TNN13880.1;TNN13881.1; | Protein cereblon isoform 2;Protein cereblon isoform 1 |
| EWB00_002587 | TNN13882.1;TNN13883.1;TNN13884.1; | RNA-binding protein isoform 2;RNA-binding protein isoform 1;RNA-binding protein isoform 3 |
| EWB00_002588 | TNN13885.1;TNN13886.1; | Vacuolar protein sorting-associated protein 41 isoform 2;Vacuolar protein sorting-associated protein 41 isoform 1 |
| EWB00_003258 | TNN13006.1;TNN13007.1;TNN13008.1; | hypothetical protein;hypothetical protein;hypothetical protein; |
| EWB00_003259 | TNN13009.1;TNN13010.1; | Syntenin-1 isoform 1;Syntenin-1 isoform 2 |
| EWB00_004024 | TNN20442.1;TNN20443.1;TNN20444.1;TNN20445.1;TNN20446.1;TNN20447.1; | Suppressor of hairless protein isoform 1;Suppressor of hairless protein isoform 2;Suppressor of hairless protein isoform 3;Suppressor of hairless protein isoform 4;Suppressor of hairless protein isoform 5 |
| EWB00_004025 | TNN20448.1; | Matrix metalloproteinase-15 |
| EWB00_004026 | TNN20449.1 | S-adenosylmethionine mitochondrial carrier protein |
| EWB00_004027 | TNN20450.1 | hypothetical protein |
| EWB00_005773 | TNN10005.1; | WD repeat-containing protein |
| EWB00_005774 |  | tRNA-OTHER |
| EWB00_005775 | TNN10006.1; | Transmembrane protein |
| EWB00_005776 | TNN10007.1; | hypothetical protein |
| EWB00_007769 | TNN07338.1; | hypothetical protein; |
| EWB00_007770 | TNN07339.1; | Long-chain-fatty-acid--CoA ligase 5 |
| EWB00_007771 | TNN07340.1; | putative Transmembrane protein |
| EWB00_007772 | TNN07341.1;TNN07342.1;TNN07343.1;TNN07344.1;TNN07345.1;TNN07346.1; | ADP-ribosylation factor-related protein isoform 2;ADP-ribosylation factor-related protein isoform 1 |
| EWB00_008227 | TNN06688.1;TNN06689.1 | Zinc/cadmium resistance protein isoform 1;Zinc/cadmium resistance protein isoform 2 |
| EWB00_008228 | TNN06690.1; | Peptidyl-prolyl cis-trans isomerase D |
| EWB00_008229 | TNN06691.1;TNN06692.1; | Synaptojanin-1 isoform 1;Synaptojanin-1 isoform 2 |
| EWB00_008461 | TNN06285.1 | Tubulin beta-4 chain |
| EWB00_008462 | TNN06286.1;TNN06287.1;TNN06288.1; | Tubulin beta chain isoform 2;Tubulin beta chain isoform 1; |
| EWB00_008463 | TNN06289.1 | Polycystin-2 |
| EWB00_008464 | TNN06290.1;TNN06291.1;TNN06292.1;TNN06293.1; | GATA-binding factor A isoform 1;GATA-binding factor A isoform 2;GATA-binding factor A isoform 3 |
| EWB00_008465 | TNN06294.1; | Proteasomal ubiquitin receptor ADRM1 |
| EWB00_008466 | TNN06295.1;TNN06296.1;TNN06297.1; | Protoporphyrinogen oxidase |
| EWB00_008467 | TNN06298.1; | Zinc finger protein |
| EWB00_008909 | TNN19435.1; | coiled-coil domain-containing 87-like isoform X1 |
| EWB00_008910 | TNN19436.1; | F-box only protein |
| EWB00_008911 | TNN19437.1;TNN19438.1;TNN19439.1;TNN19440.1;TNN19441.1;TNN19442.1; | Prefoldin subunit 6 |
| EWB00_008912 | TNN19443.1; | Transport and Golgi organization protein 1 |
| EWB00_008913 | TNN19444.1;TNN19445.1;TNN19446.1; | SPT2-like protein isoform 1;SPT2-like protein isoform 2;SPT2-like protein isoform 3 |
| EWB00_009381 | TNN19154.1; | Coiled-coil domain-containing protein |
| EWB00_009382 | TNN19155.1;TNN19156.1;TNN19157.1;TNN19158.1;TNN19159.1; | Eukaryotic translation initiation factor 2D isoform 1;Eukaryotic translation initiation factor 2D isoform 3;Eukaryotic translation initiation factor 2D isoform 2 |
| EWB00_009383 | TNN19160.1; | Adenosylhomocysteinase A |
| EWB00_009384 | TNN19161.1; | hypothetical protein |
| EWB00_009385 | TNN19162.1; | MKL/myocardin-like protein |
| EWB00_009406 | TNN19199.1 | phosphatase 3 regulatory subunit |
| EWB00_009407 | TNN19201.1;TNN19202.1; | hypothetical protein;hypothetical protein; |
| EWB00_009408 | TNN19203.1; | Glutamate receptor ionotropic, NMDA 2A |
| EWB00_009409 |  | tRNA-OTHER |
| EWB00_009573 | TNN05182.1; | Structure-specific endonuclease subunit SLX1 |
| EWB00_009574 | TNN05183.1; | Thioredoxin-like protein |
| EWB00_009575 | TNN05184.1; | Calcium-binding mitochondrial carrier protein |
| EWB00_009576 | TNN05185.1;TNN05186.1; | Protein tyrosine phosphatase domain-containing protein isoform 1;Protein tyrosine phosphatase domain-containing protein isoform 2 |
| EWB00_009818 | TNN18792.1;TNN18793.1;TNN18794.1;TNN18795.1;TNN18796.1;TNN18797.1; | putative aminopeptidase W07G4.4 isoform 1;putative aminopeptidase W07G4.4 isoform 2;putative aminopeptidase W07G4.4 isoform 3;putative aminopeptidase W07G4.4 isoform 4;putative aminopeptidase W07G4.4 isoform 5 |
| EWB00_009819 | TNN18798.1;TNN18799.1; | Dedicator of cytokinesis protein isoform 1;Dedicator of cytokinesis protein isoform 2 |
| EWB00_009820 | TNN18800.1 | hypothetical protein |
| EWB00_010824 | TNN17753.1; | hypothetical protein; |
| EWB00_010825 | TNN17754.1; | hypothetical protein; |
| EWB00_010826 | TNN17755.1; | hypothetical protein |
| EWB00_010827 | TNN17756.1 | Centrosomal protein |
| EWB00_010828 | TNN17757.1;TNN17757.1;TNN17757.1 | Kelch-like protein isoform 1;Kelch-like protein isoform 2;Kelch-like protein isoform 3 |
| EWB00_010837 | TNN17778.1 | hypothetical protein |
| EWB00_010838 | TNN17782.1 | WD repeat-containing protein |
| EWB00_010839 | TNN17783.1; | Transcriptional enhancer factor TEF-1 |
| EWB00_010840 | TNN17784.1; | Transcriptional enhancer factor TEF-1 |
| EWB00_010841 | TNN17785.1; | hypothetical protein |
| EWB00_010970 | TNN17525.1; | Histone-lysine N-methyltransferase NSD2 |
| EWB00_010971 | TNN17526.1; | Ubiquitin-like protein |
| EWB00_010972 | TNN17527.1;TNN17528.1; | Breast cancer type 2 susceptibility protein isoform 1;Breast cancer type 2 susceptibility protein isoform 2 |
| EWB00_010973 | TNN17529.1; | Paired box protein |
| EWB00_010976 | TNN17533.1; | Sarcolemmal membrane-associated protein |
| EWB00_010977 | TNN17534.1; | hypothetical protein |
| EWB00_010978 | TNN17535.1;TNN17536.1; | Mothers against decapentaplegic member 3 isoform 1;Mothers against decapentaplegic member 3 isoform 2 |
| EWB00_011050 | TNN17644.1 | Transcriptional regulator ATRX |
| EWB00_011051 | TNN17645.1 | Neurobeachin |
