# Supplementary material for: Genetic difference between two Schistosoma japonicum isolates with contrasting cercarial shedding patterns revealed by whole genome sequencing
Source: Parasite. 2023 Dec 12;30:59. doi: 10.1051/parasite/2023061 (PMC10714679; doi:10.1051/parasite/2023061)
Supplement: Supplementary file 1 — Table S1: Genes of select regions in S. japonicum from ST. Table S2: Genes of select regions in S. japonicum from HX. Table S3: GO enrichment results in S. japonicum from ST. Figure S1: Box plots of (A) π ratio (πST/πHX), (B) FST, and (C) Tajima’s D of selection regions throughout genomes. Figure S2: Fisher’s exact test (A, B) and CMH test (C, D) were employed to investigate any significant SNP frequency changes (−log10[p-value]) between the two groups. −log10(P) values for common SNPs obtained through performing FET and CMH tests were plotted together (on separate axes) in order to identify significant allele frequency changes of variants in both tests (E, F). Tests between HX2013 and ST2013 are shown in (A, C, and E), while tests between ST2013 and ST2020 are shown in (B, D, and F). The red lines represent genome-wide Bonferroni’s correction of p-value. Dots in red represent SNPs of enriched genes above the genome-wide Bonferroni’s correction level. Figure S3: FST values for entire genes calculated between HX2013 and ST2013 (A), between ST2013 and ST2020 (B), and between ST and HX (C). Dots in red represent SNPs of enriched genes. [file parasite-30-59-s1.zip › Table S2.docx]

Table S2. Genes of selection regions in *S. japonicum* from HX.

| Gene_ID | Protein_ID | Product |
| --- | --- | --- |
| EWB00_000685 | TNN16161.1; | MAGUK p55 subfamily member 7; |
| EWB00_000686 | TNN16162.1; | Protein zyg-11 isoform 1; |
| EWB00_000687 | TNN16164.1; | Chaperone protein isoform 3; |
| EWB00_000688 | TNN16169.1; | Small glutamine-rich tetratricopeptide repeat-containing protein; |
| EWB00_000689 | TNN16170.1; | Acetylcholine receptor subunit alpha-L1; |
| EWB00_000690 | TNN16171.1; | ATP-dependent RNA helicase DDX50; |
| EWB00_000691 | TNN16172.1; | Cytochrome b5 type B; |
| EWB00_000692 | TNN16173.1; | 39S ribosomal protein L37; |
| EWB00_000693 | TNN16176.1; | ataxia telangiectasia mutated (atm) isoform 2; |
| EWB00_000694 | TNN16177.1; | Serine-protein kinase ATM; |
| EWB00_000695 | TNN16178.1; | Homeobox protein; |
| EWB00_000696 | TNN16179.1; | Transcriptional enhancer factor TEF-4; |
| EWB00_000697 | TNN16180.1; | Transcription factor AP-2-epsilon; |
| EWB00_002036 | TNN20896.1; | hypothetical protein; |
| EWB00_002037 | TNN20897.1; | Asparagine synthetase domain-containing protein; |
| EWB00_002038 | TNN20898.1; | Neuropeptide F receptor; |
| EWB00_002039 | TNN20899.1; | Asparagine synthetase domain-containing protein; |
| EWB00_002191 | TNN14404.1; | hypothetical protein; |
| EWB00_002192 | TNN14405.1; | Zinc finger SWIM domain-containing protein; |
| EWB00_002193 | TNN14406.1; | Zinc finger SWIM domain-containing protein; |
| EWB00_003072 | TNN13266.1; | putative glutamate synthase [NADPH]; |
| EWB00_003073 |  | tRNA-OTHER; |
| EWB00_003074 | TNN13268.1;TNN13267.1; | NADP-dependent malic enzyme isoform 1;NADP-dependent malic enzyme isoform 2; |
| EWB00_003075 | TNN13270.1;TNN13269.1; | PR domain zinc finger protein isoform 1;PR domain zinc finger protein isoform 2; |
| EWB00_003076 | TNN13271.1; | Protein FEV; |
| EWB00_003077 |  | tRNA-OTHER; |
| EWB00_003078 | TNN13272.1; | NACHT and WD repeat domain-containing 2-like isoform 1; |
| EWB00_003079 | TNN13274.1; | Xylosyltransferase oxt; |
| EWB00_003080 | TNN13276.1;TNN13275.1;TNN13277.1;TNN13278.1;TNN13279.1; | Thioredoxin domain-containing protein isoform 3;Thioredoxin domain-containing protein isoform 1;Thioredoxin domain-containing protein isoform 4;Thioredoxin domain-containing protein isoform 2;Thioredoxin domain-containing protein isoform 5; |
| EWB00_003081 | TNN13280.1; | Glycine dehydrogenase (decarboxylating); |
| EWB00_003693 | TNN12452.1; | Two pore calcium channel protein; |
| EWB00_003694 | TNN12453.1; | Universal stress protein; |
| EWB00_003695 | TNN12459.1;TNN12457.1;TNN12456.1;TNN12460.1;TNN12458.1;TNN12454.1; | Ceramide-1-phosphate transfer protein isoform 5;Ceramide-1-phosphate transfer protein isoform 4;Ceramide-1-phosphate transfer protein isoform 5;Ceramide-1-phosphate transfer protein isoform 5;Ceramide-1-phosphate transfer protein isoform 1;Ceramide-1-phosphate transfer protein isoform 2;Ceramide-1-phosphate transfer protein isoform 3; |
| EWB00_003696 | TNN12461.1; | TM2 domain-containing protein; |
| EWB00_003697 | TNN12462.1; | Nuclear receptor corepressor 1; |
| EWB00_003784 | TNN12380.1;TNN12379.1;TNN12378.1; | UV excision repair protein RAD23 isoform 3;UV excision repair protein RAD23 isoform 2;UV excision repair protein RAD23 isoform 1; |
| EWB00_004014 | TNN20423.1;TNN20424.1;TNN20425.1; | Ras and EF-hand domain-containing protein isoform 1;Ras and EF-hand domain-containing protein isoform 2;Ras and EF-hand domain-containing protein isoform 3; |
| EWB00_004015 | TNN20428.1;TNN20427.1;TNN20426.1; | Protein YIPF6 isoform 3;Protein YIPF6 isoform 2;Protein YIPF6 isoform 1; |
| EWB00_004016 | TNN20429.1; | Lissencephaly-1 isoform 1; |
| EWB00_004017 | TNN20432.1; | hypothetical protein; |
| EWB00_004018 | TNN20433.1; | Acetyl-CoA acetyltransferase, cytosolic; |
| EWB00_004019 | TNN20434.1; | Golgin subfamily A member 7; |
| EWB00_004020 | TNN20435.1; | hypothetical protein; |
| EWB00_004021 | TNN20436.1;TNN20439.1;TNN20438.1;TNN20437.1; | Caseinolytic peptidase B protein isoform 2;Caseinolytic peptidase B protein isoform 4;Caseinolytic peptidase B protein isoform 1;Caseinolytic peptidase B protein isoform 3; |
| EWB00_004022 | TNN20440.1; | STAM-binding protein; |
| EWB00_004023 | TNN20441.1; | Ubiquitin-like-conjugating enzyme ATG10; |
| EWB00_004024 | TNN20446.1;TNN20443.1;TNN20442.1;TNN20447.1;TNN20445.1; | Suppressor of hairless protein isoform 1;Suppressor of hairless protein isoform 2;Suppressor of hairless protein isoform 3;Suppressor of hairless protein isoform 4;Suppressor of hairless protein isoform 5; |
| EWB00_004630 | TNN11361.1; | Acetylcholine receptor subunit alpha-L1; |
| EWB00_004631 | TNN11362.1;TNN11363.1; | nachr subunit isoform 1;nachr subunit isoform 2; |
| EWB00_004632 | TNN11364.1; | Vacuolar protein sorting-associated protein 72; |
| EWB00_004633 | TNN11365.1; | NADH dehydrogenase [ubiquinone] iron-sulfur protein 4; |
| EWB00_004634 | TNN11366.1; | Cytospin-A; |
| EWB00_004635 | TNN11367.1; | Doublesex and mab-3 related transcription factor 3; |
| EWB00_004636 | TNN11368.1; | hypothetical protein; |
| EWB00_004637 | TNN11369.1; | Ran-binding protein; |
| EWB00_004638 | TNN11370.1; | hypothetical protein; |
| EWB00_004639 | TNN11371.1; | DNA-directed RNA polymerases RPABC4; |
| EWB00_004665 | TNN21411.1; | Small G protein signaling modulator 3; |
| EWB00_004666 | TNN21412.1;TNN21413.1;TNN21414.1;TNN21415.1; | hypothetical protein;hypothetical protein;hypothetical protein;hypothetical protein; |
| EWB00_004667 | TNN21417.1;TNN21416.1; | Negative elongation factor D isoform 2;Negative elongation factor D isoform 1; |
| EWB00_004668 | TNN21418.1;TNN21419.1; | Cilia- and flagella-associated protein isoform 1;Cilia- and flagella-associated protein isoform 2; |
| EWB00_004671 | TNN21422.1; | sodium dicarboxylate cotransporter-related; |
| EWB00_004672 | TNN21423.1;TNN21424.1;TNN21425.1; | Solute carrier family 13 member 2 isoform 1;Solute carrier family 13 member 2 isoform 2;Solute carrier family 13 member 2 isoform 1; |
| EWB00_004682 | TNN21442.1; | Frizzled-10-A; |
| EWB00_004683 | TNN21443.1; | Macoilin-1; |
| EWB00_004684 | TNN21444.1; | General transcription factor 3C polypeptide 3; |
| EWB00_005056 | TNN10837.1;TNN10838.1;TNN10839.1;TNN10840.1;TNN10841.1; | hypothetical protein;hypothetical protein;hypothetical protein;hypothetical protein;hypothetical protein; |
| EWB00_005057 | TNN10842.1; | Protein FAM76A; |
| EWB00_005058 | TNN10843.1;TNN10844.1;TNN10845.1;TNN10846.1;TNN10847.1;TNN10848.1; | Rho guanine nucleotide exchange factor 7 isoform 1;Rho guanine nucleotide exchange factor 7 isoform 2;Rho guanine nucleotide exchange factor 7 isoform 3;Rho guanine nucleotide exchange factor 7 isoform 4;Rho guanine nucleotide exchange factor 7 isoform 5;Rho guanine nucleotide exchange factor 7 isoform 6; |
| EWB00_005059 | TNN10849.1; | [Pyruvate dehydrogenase [acetyl-transferring]]-phosphatase 2; |
| EWB00_005495 | TNN10333.1; | ARL14 effector; |
| EWB00_005496 | TNN10334.1;TNN10335.1; | Nucleotidyltransferase isoform 1;Nucleotidyltransferase isoform 2; |
| EWB00_005497 | TNN10336.1;TNN10337.1;TNN10338.1;TNN10339.1; | hypothetical protein;hypothetical protein;hypothetical protein;hypothetical protein; |
| EWB00_005546 | TNN10282.1; | Netrin receptor UNC5B; |
| EWB00_005547 | TNN10283.1; | Sperm-tail PG-rich repeat-containing protein; |
| EWB00_005548 | TNN10285.1;TNN10284.1; | hypothetical protein;hypothetical protein; |
| EWB00_005549 | TNN10286.1; | Protein phosphatase ppm-1; |
| EWB00_005550 | TNN10287.1; | Protein ABHD4; |
| EWB00_005551 | TNN10288.1; | Protein ABHD4; |
| EWB00_005769 | TNN10060.1; | PHD finger protein; |
| EWB00_005770 | TNN10061.1; | putative glutamate receptor; |
| EWB00_005771 | TNN10062.1; | hypothetical protein; |
| EWB00_005772 | TNN10063.1;TNN10064.1;TNN10065.1;TNN10066.1;TNN10067.1;TNN10068.1; | Methyltransferase-like protein 17 isoform 1;Methyltransferase-like protein 17 isoform 2;Methyltransferase-like protein 17 isoform 3;Methyltransferase-like protein 17 isoform 4;Methyltransferase-like protein 17 isoform 5;Methyltransferase-like protein 17 isoform 5; |
| EWB00_006005 | TNN19794.1;TNN19795.1;TNN19796.1;TNN19797.1; | Thymidylate synthase isoform 1;Thymidylate synthase isoform 2;Thymidylate synthase isoform 2;Thymidylate synthase isoform 3; |
| EWB00_006006 | TNN19798.1; | hypothetical protein; |
| EWB00_006006 | TNN19798.1; | hypothetical protein; |
| EWB00_006008 | TNN19802.1; | polymerase delta-interacting protein; |
| EWB00_006009 | TNN19803.1;TNN19804.1;TNN19805.1;TNN19806.1; | Inositol-tetrakisphosphate 1-kinase isoform 1;Inositol-tetrakisphosphate 1-kinase isoform 2;Inositol-tetrakisphosphate 1-kinase isoform 3;Inositol-tetrakisphosphate 1-kinase isoform 3; |
| EWB00_006010 | TNN19807.1; | H/ACA ribonucleoprotein complex subunit 2; |
| EWB00_006011 | TNN19808.1; | hypothetical protein; |
| EWB00_006012 | TNN19809.1; | Autophagy-related protein; |
| EWB00_006013 | TNN19810.1; | U6 snRNA-associated Sm-like protein; |
| EWB00_006014 | TNN19812.1;TNN19813.1;TNN19811.1; | UPF0160 protein MYG1 isoform 2;UPF0160 protein MYG1 isoform 2;UPF0160 protein MYG1 isoform 1; |
| EWB00_007527 | TNN07773.1; | 28S ribosomal protein S17; |
| EWB00_007528 | TNN07774.1; | hypothetical protein; |
| EWB00_007529 | TNN07775.1; | hypothetical protein; |
| EWB00_007530 | TNN07776.1; | hypothetical protein; |
| EWB00_007531 | TNN07777.1; | hypothetical protein; |
| EWB00_007532 | TNN07778.1; | Phosphatidylinositol transfer protein; |
| EWB00_007533 | TNN07779.1; | Spondin-1; |
| EWB00_007534 | TNN07780.1; | TATA-binding protein-associated factor; |
| EWB00_007535 | TNN07781.1; | TATA-binding protein-associated factor; |
| EWB00_007536 | TNN07783.1;TNN07782.1; | Dolichyl-diphosphooligosaccharide--protein glycosyltransferase subunit STT3B; |
| EWB00_007537 |  | tRNA-OTHER; |
| EWB00_007782 | TNN07360.1; | Set1/Ash2 histone methyltransferase complex subunit ASH2; |
| EWB00_007783 | TNN07361.1;TNN07362.1; | Poly(ADP-ribose) glycohydrolase isoform 1;Poly(ADP-ribose) glycohydrolase isoform 2; |
| EWB00_007784 | TNN07364.1;TNN07363.1; | Laminin subunit alpha-2 isoform 2;Laminin subunit alpha-2 isoform 2; |
| EWB00_007785 | TNN07365.1; | hypothetical protein; |
| EWB00_007786 | TNN07366.1; | short chain dehydrogenase; |
| EWB00_007787 | TNN07367.1; | Dehydrogenase/reductase SDR family member 1; |
| EWB00_007788 | TNN07370.1;TNN07369.1;TNN07368.1; | Dehydrogenase/reductase SDR family member 1 isoform 2;Dehydrogenase/reductase SDR family member 1 isoform 1;Dehydrogenase/reductase SDR family member 1 isoform 1; |
| EWB00_007789 | TNN07371.1; | Protein yippee-like 5; |
| EWB00_007790 | TNN07372.1;TNN07373.1;TNN07374.1; | putative hydroxyacylglutathione hydrolase isoform 1;putative hydroxyacylglutathione hydrolase isoform 2;putative hydroxyacylglutathione hydrolase isoform 2; |
| EWB00_007791 | TNN07377.1;TNN07376.1;TNN07375.1; | putative yippee protein isoform 3;putative yippee protein isoform 2;putative yippee protein isoform 1; |
| EWB00_007953 | TNN07074.1; | Zinc transporter ZIP13; |
| EWB00_007954 | TNN07075.1;TNN07076.1;TNN07077.1;TNN07078.1; | hypothetical protein;hypothetical protein;hypothetical protein;hypothetical protein; |
| EWB00_007955 | TNN07079.1; | CIP2A; |
| EWB00_007956 | TNN07081.1;TNN07080.1; | G2/mitotic-specific cyclin-B2 isoform 2;G2/mitotic-specific cyclin-B2 isoform 1; |
| EWB00_007957 | TNN07084.1;TNN07083.1;TNN07082.1; | putative 18S rRNA (guanine-N(7))-methyltransferase isoform 1;putative 18S rRNA (guanine-N(7))-methyltransferase isoform 2;putative 18S rRNA (guanine-N(7))-methyltransferase isoform 2; |
| EWB00_007958 | TNN07085.1;TNN07086.1; | Transcription initiation factor TFIID subunit 1 isoform 1;Transcription initiation factor TFIID subunit 1 isoform 2; |
| EWB00_008262 | TNN06610.1; | rhodopsin-like orphan GPCR; |
| EWB00_008263 | TNN06611.1; | rhodopsin-like orphan GPCR; |
| EWB00_008264 | TNN06612.1; | rhodopsin-like orphan GPCR; |
| EWB00_008265 | TNN06613.1;TNN06614.1;TNN06615.1; | Wiskott-Aldrich syndrome protein family member 3 isoform 2;Wiskott-Aldrich syndrome protein family member 3 isoform 1;Wiskott-Aldrich syndrome protein family member 3 isoform 2; |
| EWB00_008266 | TNN06616.1;TNN06617.1;TNN06618.1; | hypothetical protein;hypothetical protein;hypothetical protein; |
| EWB00_008267 | TNN06619.1; | hypothetical protein; |
| EWB00_008325 | TNN06530.1;TNN06531.1;TNN06529.1; | adenylate cyclase isoform 1;adenylate cyclase isoform 3;adenylate cyclase isoform 2; |
| EWB00_008326 | TNN06533.1;TNN06532.1; | adenylate cyclase isoform 1;adenylate cyclase isoform 1; |
| EWB00_008327 | TNN06534.1;TNN06535.1; | adenylate cyclase; |
| EWB00_008328 | TNN06536.1; | adenylate cyclase; |
| EWB00_008329 | TNN06538.1;TNN06539.1;TNN06537.1; | adenylate cyclase isoform 1;adenylate cyclase isoform 1;adenylate cyclase isoform 2; |
| EWB00_008330 | TNN06540.1;TNN06541.1;TNN06542.1;TNN06543.1; | adenylate cyclase isoform 1;adenylate cyclase isoform 2;adenylate cyclase isoform 3;adenylate cyclase isoform 4; |
| EWB00_008438 | TNN06313.1;TNN06314.1;TNN06315.1;TNN06316.1;TNN06317.1; | hypothetical protein;hypothetical protein;hypothetical protein;hypothetical protein;hypothetical protein; |
| EWB00_008439 | TNN06318.1; | hypothetical protein; |
| EWB00_008440 | TNN06319.1; | clasp1 cytoplasmic linker associated protein; |
| EWB00_008441 |  | tRNA-OTHER; |
| EWB00_008442 | TNN06320.1; | putative multiple ankyrin repeat-containing single kh domain protein; |
| EWB00_008443 | TNN06321.1; | Ankyrin repeat and KH domain-containing protein; |
| EWB00_008444 | TNN06322.1; | Protein EFR3A; |
| EWB00_008446 | TNN06325.1;TNN06326.1;TNN06327.1;TNN06328.1;TNN06329.1; | Moesin ezrin radixin 1 isoform 1;Moesin ezrin radixin 1 isoform 2;Moesin ezrin radixin 1 isoform 1;Moesin ezrin radixin 1 isoform 3;Moesin ezrin radixin 1 isoform 3; |
| EWB00_008447 | TNN06330.1;TNN06331.1;TNN06332.1;TNN06333.1;TNN06334.1;TNN06335.1;TNN06336.1;TNN06337.1; | hypothetical protein; |
| EWB00_008448 | TNN06338.1; | Cytochrome c oxidase assembly protein COX15; |
| EWB00_008449 | TNN06339.1; | Protein VHS3; |
| EWB00_008450 | TNN06340.1; | nucleolin; |
| EWB00_008451 | TNN06341.1;TNN06342.1; | E3 ubiquitin-protein ligase TRIM37 isoform 1;E3 ubiquitin-protein ligase TRIM37 isoform 2; |
| EWB00_008452 | TNN06343.1; | hypothetical protein; |
| EWB00_008455 | TNN19544.1; | Proteasome-associated protein ECM29; |
| EWB00_008456 | TNN19545.1;TNN19546.1; | KH domain-containing, RNA-binding, signal transduction-associated protein isoform 1;KH domain-containing, RNA-binding, signal transduction-associated protein isoform 2; |
| EWB00_008457 | TNN19547.1; | XK-related protein; |
| EWB00_008458 | TNN19548.1; | CD97 antigen; |
| EWB00_008459 | TNN19549.1; | Protein tilB; |
| EWB00_008502 | TNN06227.1; | Dynein heavy chain 5, axonemal; |
| EWB00_008503 | TNN06228.1; | Gag-Pol polyprotein; |
| EWB00_008504 |  | tRNA-OTHER; |
| EWB00_008505 |  | tRNA-OTHER; |
| EWB00_008506 | TNN06231.1; | Mitochondrial import inner membrane translocase subunit Tim23 isoform 1; |
| EWB00_008507 | TNN06235.1; | Protein lozenge; |
| EWB00_008508 | TNN06236.1;TNN06237.1;TNN06238.1; | Eukaryotic translation initiation factor 2 subunit 3 isoform 1;Eukaryotic translation initiation factor 2 subunit 3 isoform 2;Eukaryotic translation initiation factor 2 subunit 3 isoform 3; |
| EWB00_008509 | TNN06239.1; | THUMP domain-containing protein 1; |
| EWB00_008510 | TNN06240.1;TNN06241.1; | Porphobilinogen deaminase isoform 1;Porphobilinogen deaminase isoform 2; |
| EWB00_008511 | TNN06242.1;TNN06243.1; | hypothetical protein;hypothetical protein; |
| EWB00_008512 | TNN06244.1; | putative atp-binding cassette transporter; |
| EWB00_008513 | TNN06245.1;TNN06246.1;TNN06247.1;TNN06248.1;TNN06249.1; | Superkiller viralicidic activity 2-like 2 isoform 3;Superkiller viralicidic activity 2-like 2 isoform 2;Superkiller viralicidic activity 2-like 2 isoform 1;Superkiller viralicidic activity 2-like 2 isoform 1;Superkiller viralicidic activity 2-like 2 isoform 3; |
| EWB00_008514 | TNN06250.1;TNN06251.1; | NADH dehydrogenase [ubiquinone] iron-sulfur protein 3; |
| EWB00_008515 | TNN06252.1; | hypothetical protein; |
| EWB00_008590 | TNN06101.1; | Retrovirus-related Pol polyprotein; |
| EWB00_008591 | TNN06102.1; | Splicing factor U2AF 65 kDa subunit; |
| EWB00_008592 | TNN06103.1;TNN06104.1; | WD repeat-containing protein isoform 1;WD repeat-containing protein isoform 2; |
| EWB00_008593 | TNN06106.1;TNN06105.1;TNN06107.1; | hypothetical protein;hypothetical protein;hypothetical protein; |
| EWB00_008594 | TNN06108.1; | Endoglin antigen; |
| EWB00_008595 | TNN06109.1;TNN06110.1; | Calcyphosin-like protein isoform 1;Calcyphosin-like protein isoform 2; |
| EWB00_008596 | TNN06111.1; | Aminoacylase-1; |
| EWB00_008597 | TNN06112.1; | Kinesin-associated protein; |
| EWB00_008598 | TNN06113.1;TNN06114.1;TNN06115.1; | Diacylglycerol O-acyltransferase 1 isoform 1;Diacylglycerol O-acyltransferase 1 isoform 3;Diacylglycerol O-acyltransferase 1 isoform 2; |
| EWB00_008660 | TNN06028.1; | General transcription factor IIH subunit 1; |
| EWB00_008661 | TNN06029.1;TNN06030.1; | Beta-galactosidase-1-like protein isoform 1;Beta-galactosidase-1-like protein isoform 2; |
| EWB00_008662 | TNN06033.1;TNN06032.1;TNN06031.1; | Neuronal acetylcholine receptor subunit alpha-6 isoform 3;Neuronal acetylcholine receptor subunit alpha-6 isoform 1;Neuronal acetylcholine receptor subunit alpha-6 isoform 2; |
| EWB00_008663 | TNN06034.1; | Pikachurin; |
| EWB00_008664 | TNN06037.1;TNN06036.1;TNN06035.1;TNN06038.1; | Zinc finger protein isoform 1;Zinc finger protein isoform 2; |
| EWB00_008665 | TNN06039.1; | endonuclease reverse transcriptase; |
| EWB00_008704 | TNN05981.1; | E3 ubiquitin- ligase ICP0; |
| EWB00_008705 | TNN05983.1;TNN05982.1;TNN05984.1; | Replication protein A 32 kDa subunit isoform 2;Replication protein A 32 kDa subunit isoform 1;Replication protein A 32 kDa subunit isoform 3; |
| EWB00_008706 | TNN05985.1;TNN05986.1; | Peptidyl-prolyl cis-trans isomerase-like 4 isoform 1;Peptidyl-prolyl cis-trans isomerase-like 4 isoform 2; |
| EWB00_008707 | TNN05987.1; | hypothetical protein; |
| EWB00_008708 | TNN05988.1; | hypothetical protein; |
| EWB00_008709 | TNN05989.1;TNN05990.1;TNN05991.1;TNN05992.1;TNN05993.1; | E3 ubiquitin-protein ligase listerin isoform 1;E3 ubiquitin-protein ligase listerin isoform 2;E3 ubiquitin-protein ligase listerin isoform 3;E3 ubiquitin-protein ligase listerin isoform 4;E3 ubiquitin-protein ligase listerin isoform 5; |
| EWB00_008710 | TNN05996.1;TNN05994.1;TNN05995.1; | hypothetical protein; |
| EWB00_008838 | TNN05824.1; | Forkhead box protein; |
| EWB00_008839 | TNN05827.1;TNN05826.1;TNN05825.1; | Calcium-responsive transactivator; |
| EWB00_008840 | TNN05828.1;TNN05829.1;TNN05830.1;TNN05831.1; | E3 ubiquitin-protein ligase PDZRN3-B isoform 1;E3 ubiquitin-protein ligase PDZRN3-B isoform 2; |
| EWB00_008841 | TNN05832.1;TNN05833.1;TNN05834.1;TNN05835.1;TNN05836.1;TNN05837.1;TNN05838.1;TNN05839.1; | putative ribonuclease ZC3H12D isoform 1;putative ribonuclease ZC3H12D isoform 2;putative ribonuclease ZC3H12D isoform 3; |
| EWB00_008842 | TNN05840.1; | N-acetylgalactosamine kinase; |
| EWB00_008843 | TNN05841.1; | hypothetical protein; |
| EWB00_009090 | TNN05682.1; | Protein unc-80; |
| EWB00_009092 | TNN05683.1; | hypothetical protein; |
| EWB00_009093 | TNN05684.1;TNN05685.1;TNN05687.1; | Inositol-pentakisphosphate 2-kinase isoform 1;Inositol-pentakisphosphate 2-kinase isoform 2;Inositol-pentakisphosphate 2-kinase isoform 3; |
| EWB00_009094 | TNN05687.1; | Allatostatin-A receptor; |
| EWB00_009095 | TNN05688.1; | Tyrosine-protein kinase FRK; |
| EWB00_009096 |  | tRNA-OTHER; |
| EWB00_009097 | TNN05689.1;TNN05690.1; | Solute carrier family 25 member 46 isoform 1;Solute carrier family 25 member 46 isoform 2; |
| EWB00_009195 | TNN05525.1;TNN05524.1; | Heat shock factor protein; |
| EWB00_009196 | TNN05526.1;TNN05527.1;TNN05528.1;TNN05529.1; | Prolyl 4-hydroxylase subunit alpha-2 isoform 1;Prolyl 4-hydroxylase subunit alpha-2 isoform 2;Prolyl 4-hydroxylase subunit alpha-2 isoform 3;Prolyl 4-hydroxylase subunit alpha-2 isoform 4; |
| EWB00_009243 | TNN05446.1;TNN05447.1; | otopetrin-2-like isoform X1 isoform 1;otopetrin-2-like isoform X1 isoform 2; |
| EWB00_009244 | TNN05448.1;TNN05449.1; | hypothetical protein;hypothetical protein; |
| EWB00_009245 | TNN05451.1;TNN05450.1; | EGF type aspartate asparagine hydroxylation site; |
| EWB00_009246 | TNN05452.1;TNN05453.1;TNN05454.1;TNN05455.1;TNN05456.1;TNN05457.1; | Glycine cleavage system H protein isoform 1;Glycine cleavage system H protein isoform 2;Glycine cleavage system H protein isoform 3;Glycine cleavage system H protein isoform 4;Glycine cleavage system H protein isoform 5; |
| EWB00_009415 | TNN19217.1;TNN19215.1;TNN19216.1; | Beta-1,4-N-acetylgalactosaminyltransferase bre-4 isoform 1;Beta-1,4-N-acetylgalactosaminyltransferase bre-4 isoform 2; |
| EWB00_009416 | TNN19218.1;TNN19219.1; | Noggin isoform 1;Noggin isoform 2; |
| EWB00_009417 | TNN19220.1;TNN19221.1; | Cilia- and flagella-associated protein isoform 1;Cilia- and flagella-associated protein isoform 2; |
| EWB00_009418 | TNN19224.1;TNN19223.1;TNN19222.1; | hypothetical protein; |
| EWB00_009419 | TNN19225.1; | Protein timeless; |
| EWB00_009439 | TNN05278.1; | U3 small nucleolar ribonucleoprotein; |
| EWB00_009440 | TNN05279.1;TNN05280.1;TNN05281.1;TNN05282.1; | Adenylate kinase 2 isoform 1;Adenylate kinase 2 isoform 2;Adenylate kinase 2 isoform 3;Adenylate kinase 2 isoform 4; |
| EWB00_009441 | TNN05283.1; | hypothetical protein; |
| EWB00_009461 | TNN19038.1; | Beta-1,4-N-acetylgalactosaminyltransferase bre-4; |
| EWB00_009462 | TNN19039.1; | hypothetical protein; |
| EWB00_009463 | TNN19040.1; | hypothetical protein; |
| EWB00_009464 | TNN19041.1;TNN19042.1;TNN19043.1; | IQ domain-containing protein isoform 1;IQ domain-containing protein isoform 2;IQ domain-containing protein isoform 3; |
| EWB00_009465 | TNN19044.1;TNN19045.1;TNN19046.1; | Glutamine synthetase isoform 1;Glutamine synthetase isoform 2;Glutamine synthetase isoform 3; |
| EWB00_009466 | TNN19047.1; | Pol polyprotein; |
| EWB00_009467 | TNN19048.1;TNN19049.1; | hypothetical protein; |
| EWB00_009468 | TNN19050.1; | QRFP-like peptide receptor; |
| EWB00_009474 | TNN19061.1;TNN19060.1; | Cysteine-rich PDZ-binding protein isoform 1;Cysteine-rich PDZ-binding protein isoform 2; |
| EWB00_009475 | TNN19062.1; | DnaJ subfamily B member 12; |
| EWB00_009476 | TNN19063.1;TNN19065.1;TNN19064.1; | putative inositol monophosphatase 3; |
| EWB00_009477 | TNN19066.1; | myo inositol monophosphatase; |
| EWB00_009478 | TNN19067.1; | Transcription initiation factor TFIID subunit 13; |
| EWB00_009479 | TNN19068.1; | Serine/threonine-protein kinase MAK; |
| EWB00_009486 | TNN19075.1; | SUN domain-containing-like protein; |
| EWB00_009487 | TNN19076.1; | Gem-associated protein; |
| EWB00_009488 | TNN19077.1; | Protein phosphatase 1 regulatory inhibitor subunit 16B; |
| EWB00_009489 | TNN19078.1;TNN19079.1; | Mitochondrial inner membrane protein isoform 1;Mitochondrial inner membrane protein isoform 2; |
| EWB00_009490 | TNN19080.1; | Peptidyl-prolyl cis-trans isomerase E; |
| EWB00_009503 | TNN05245.1; | Solute carrier family 25 member 51; |
| EWB00_009504 | TNN05246.1; | Vacuolar fusion protein CCZ1; |
| EWB00_009505 | TNN05247.1; | Mitochondrial import inner membrane translocase subunit Tim29; |
| EWB00_009507 | TNN05234.1;TNN05235.1;TNN05236.1;TNN05237.1; | putative anion transporter 4 isoform 1;putative anion transporter 4 isoform 2; |
| EWB00_009508 | TNN05238.1;TNN05239.1;TNN05240.1;TNN05241.1;TNN05242.1; | Inositol monophosphatase 2 isoform 1;Inositol monophosphatase 2 isoform 2;Inositol monophosphatase 2 isoform 3;Inositol monophosphatase 2 isoform 4;Inositol monophosphatase 2 isoform 5; |
| EWB00_009509 | TNN05243.1; | putative Polycomb group protein; |
| EWB00_009512 | TNN05229.1; | Synaptogyrin-2; |
| EWB00_009513 | TNN05230.1; | Ufm1-specific protease 2; |
| EWB00_009514 | TNN05231.1; | Ufm1-specific protease 2; |
| EWB00_009622 | TNN05144.1;TNN05145.1; | ATP-binding cassette sub-family A member 3; |
| EWB00_009623 | TNN05146.1; | Ionotropic receptor 25a; |
| EWB00_009624 | TNN05147.1;TNN05148.1;TNN05149.1; | hypothetical protein;hypothetical protein;hypothetical protein; |
| EWB00_009638 | TNN05132.1;TNN05133.1;TNN05134.1;TNN05135.1;TNN05136.1;TNN05137.1; | Glucose-6-phosphate exchanger SLC37A2 isoform 1;Glucose-6-phosphate exchanger SLC37A2 isoform 2;Glucose-6-phosphate exchanger SLC37A2 isoform 3;Glucose-6-phosphate exchanger SLC37A2 isoform 4;Glucose-6-phosphate exchanger SLC37A2 isoform 5;Glucose-6-phosphate exchanger SLC37A2 isoform 6; |
| EWB00_009673 | TNN05102.1;TNN05103.1;TNN05104.1;TNN05105.1;TNN05106.1;TNN05107.1; | hypothetical protein; |
| EWB00_009674 | TNN05108.1; | Guanine nucleotide exchange factor MSS4; |
| EWB00_009684 | TNN05085.1;TNN05086.1;TNN05087.1;TNN05088.1;TNN05089.1;TNN05090.1;TNN05091.1; | p11 protein isoform 1;p11 protein isoform 2;p11 protein isoform 3;p11 protein isoform 4;p11 protein isoform 5; |
| EWB00_009790 | TNN18762.1; | hypothetical protein; |
| EWB00_009791 | TNN18763.1;TNN18764.1;TNN18765.1; | Suppressor of tumorigenicity 7 protein isoform 1; |
| EWB00_009792 | TNN18766.1; | Regulator of G-protein signaling 8; |
| EWB00_009793 | TNN18767.1; | Integrator complex subunit 1; |
| EWB00_009794 | TNN18768.1;TNN18769.1;TNN18770.1;TNN18771.1; | CDK5RAP3-like protein isoform 1;CDK5RAP3-like protein isoform 2;CDK5RAP3-like protein isoform 3; |
| EWB00_009795 | TNN18772.1; | Unconventional myosin-XV; |
| EWB00_009796 | TNN18773.1; | Transmembrane protein; |
| EWB00_009797 | TNN18774.1; | hypothetical protein; |
| EWB00_009798 | TNN18775.1; | Homer protein; |
| EWB00_009799 | TNN18776.1;TNN18777.1;TNN18778.1; | Exosome complex component MTR3 isoform 1;Exosome complex component MTR3 isoform 2; |
| EWB00_009800 | TNN18780.1;TNN18779.1; | E3 ubiquitin-protein ligase Ubr3 isoform 1;E3 ubiquitin-protein ligase Ubr3 isoform 2; |
| EWB00_009801 | TNN18781.1; | Photosynthetic reaction centre L M; |
| EWB00_009802 | TNN18782.1;TNN18783.1; | Regulator of G-protein signaling 7 isoform 1;Regulator of G-protein signaling 7 isoform 2; |
| EWB00_009803 | TNN18784.1; | PR domain zinc finger protein; |
| EWB00_009804 | TNN18785.1; | hypothetical protein; |
| EWB00_009805 | TNN18786.1; | PAX-interacting protein; |
| EWB00_009806 | TNN18787.1; | Homeobox protein; |
| EWB00_009918 | TNN04994.1; | hypothetical protein; |
| EWB00_009919 | TNN04995.1; | Gamma-tubulin complex component 2; |
| EWB00_009920 | TNN18237.1; | hypothetical protein; |
| EWB00_010371 | TNN18237.1; | hypothetical protein; |
| EWB00_010372 | TNN18238.1; | Retrovirus-related Pol polyprotein; |
| EWB00_010373 | TNN18239.1;TNN18240.1;TNN18241.1; | Dynein intermediate chain 3, ciliary isoform 1;Dynein intermediate chain 3, ciliary isoform 2;Dynein intermediate chain 3, ciliary isoform 3; |
| EWB00_010374 | TNN18242.1;TNN18243.1; | Alpha-1,2-mannosyltransferase ALG9; |
| EWB00_010375 | TNN18244.1;TNN18245.1; | Centrosomal protein isoform 1; |
| EWB00_011067 | TNN17672.1; | hypothetical protein; |
| EWB00_011068 | TNN17673.1; | hypothetical protein; |
