# Supplementary figures and images for: Genetic difference between two Schistosoma japonicum isolates with contrasting cercarial shedding patterns revealed by whole genome sequencing
Source: Parasite. 2023 Dec 12;30:59. doi: 10.1051/parasite/2023061 (PMC10714679; doi:10.1051/parasite/2023061)

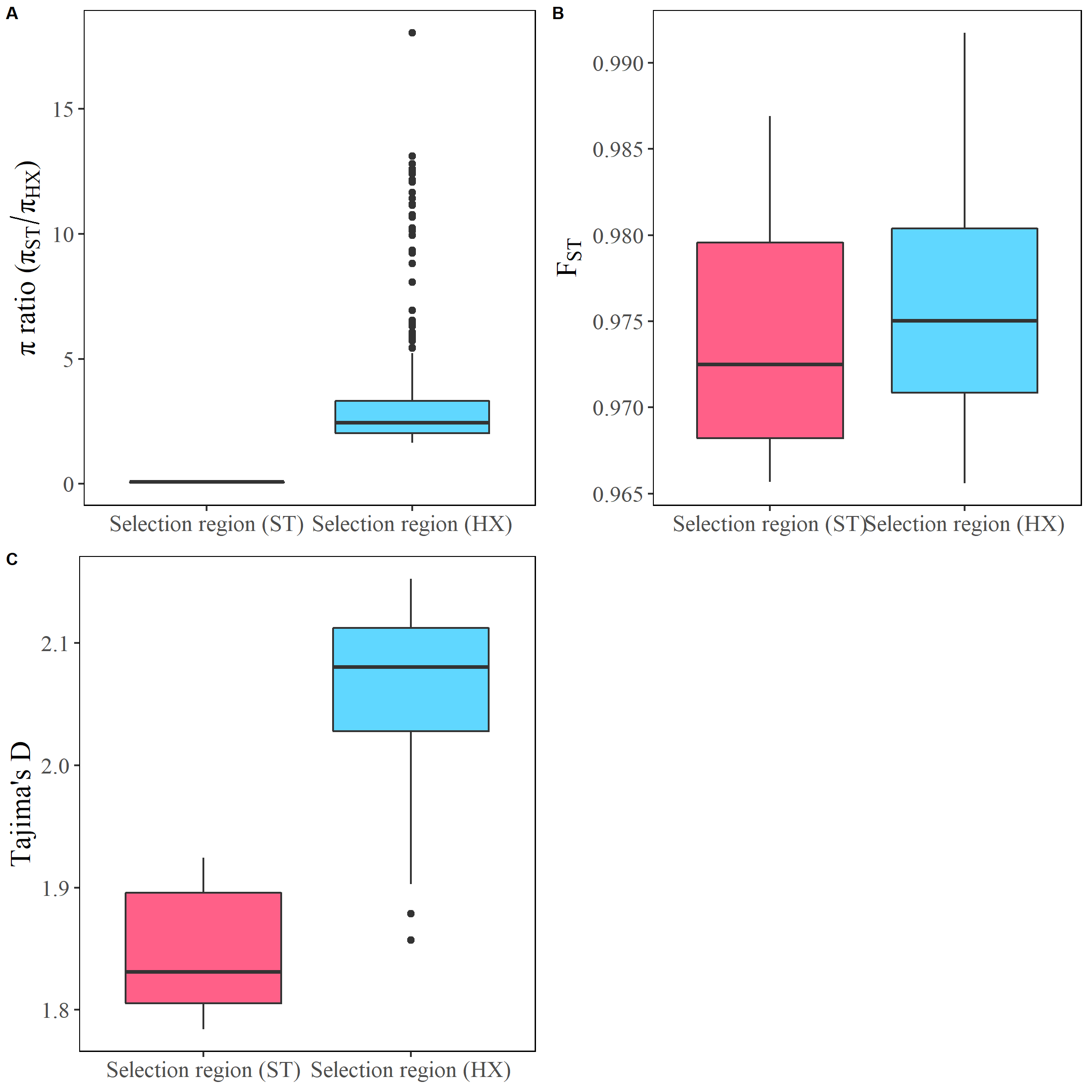

Supplement: Supplementary file 1 — Table S1: Genes of select regions in S. japonicum from ST. Table S2: Genes of select regions in S. japonicum from HX. Table S3: GO enrichment results in S. japonicum from ST. Figure S1: Box plots of (A) π ratio (πST/πHX), (B) FST, and (C) Tajima’s D of selection regions throughout genomes. Figure S2: Fisher’s exact test (A, B) and CMH test (C, D) were employed to investigate any significant SNP frequency changes (−log10[p-value]) between the two groups. −log10(P) values for common SNPs obtained through performing FET and CMH tests were plotted together (on separate axes) in order to identify significant allele frequency changes of variants in both tests (E, F). Tests between HX2013 and ST2013 are shown in (A, C, and E), while tests between ST2013 and ST2020 are shown in (B, D, and F). The red lines represent genome-wide Bonferroni’s correction of p-value. Dots in red represent SNPs of enriched genes above the genome-wide Bonferroni’s correction level. Figure S3: FST values for entire genes calculated between HX2013 and ST2013 (A), between ST2013 and ST2020 (B), and between ST and HX (C). Dots in red represent SNPs of enriched genes. [file parasite-30-59-s1.zip › Figure S1.tiff]

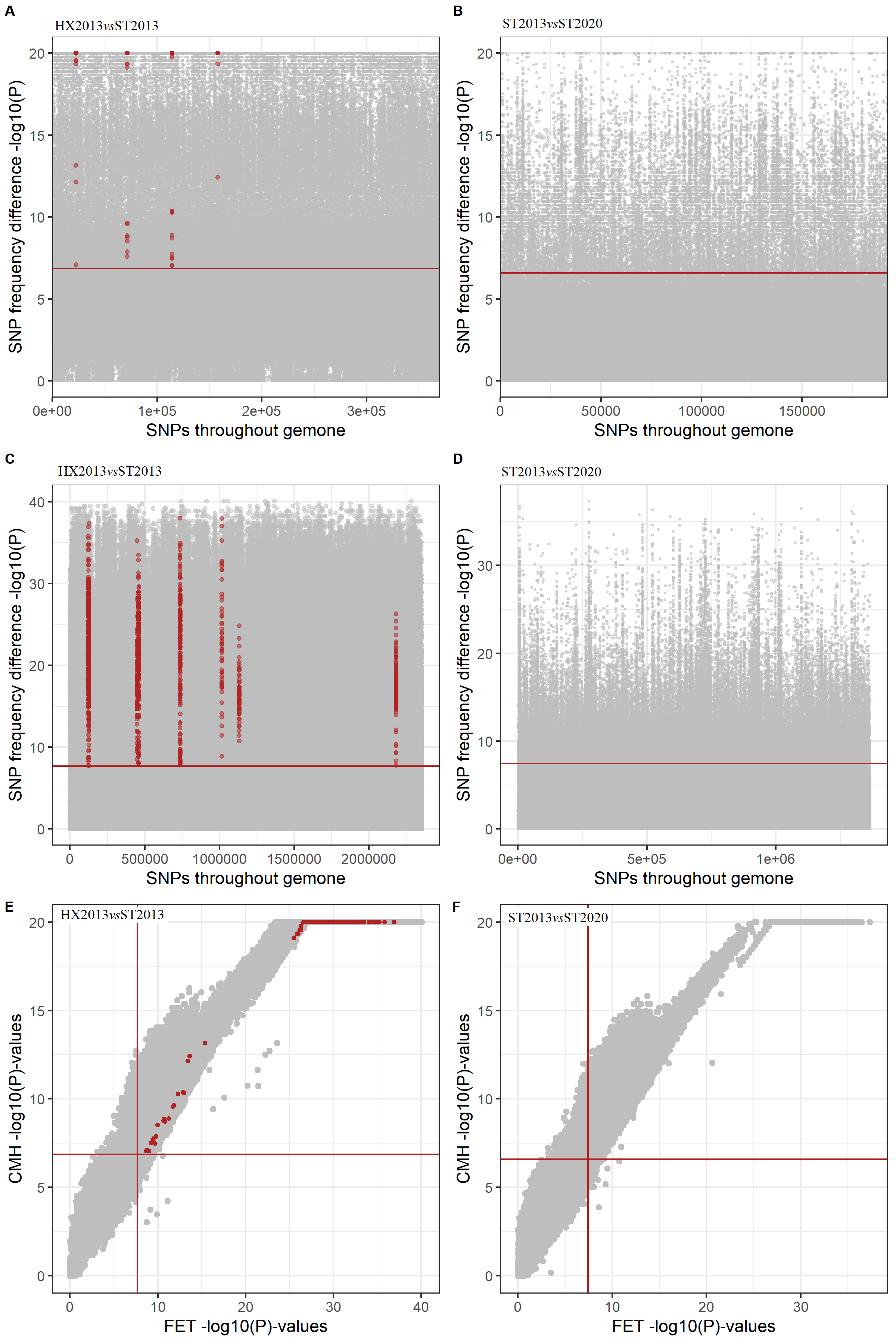

Supplement: Supplementary file 1 — Table S1: Genes of select regions in S. japonicum from ST. Table S2: Genes of select regions in S. japonicum from HX. Table S3: GO enrichment results in S. japonicum from ST. Figure S1: Box plots of (A) π ratio (πST/πHX), (B) FST, and (C) Tajima’s D of selection regions throughout genomes. Figure S2: Fisher’s exact test (A, B) and CMH test (C, D) were employed to investigate any significant SNP frequency changes (−log10[p-value]) between the two groups. −log10(P) values for common SNPs obtained through performing FET and CMH tests were plotted together (on separate axes) in order to identify significant allele frequency changes of variants in both tests (E, F). Tests between HX2013 and ST2013 are shown in (A, C, and E), while tests between ST2013 and ST2020 are shown in (B, D, and F). The red lines represent genome-wide Bonferroni’s correction of p-value. Dots in red represent SNPs of enriched genes above the genome-wide Bonferroni’s correction level. Figure S3: FST values for entire genes calculated between HX2013 and ST2013 (A), between ST2013 and ST2020 (B), and between ST and HX (C). Dots in red represent SNPs of enriched genes. [file parasite-30-59-s1.zip › Figure S2.tiff]

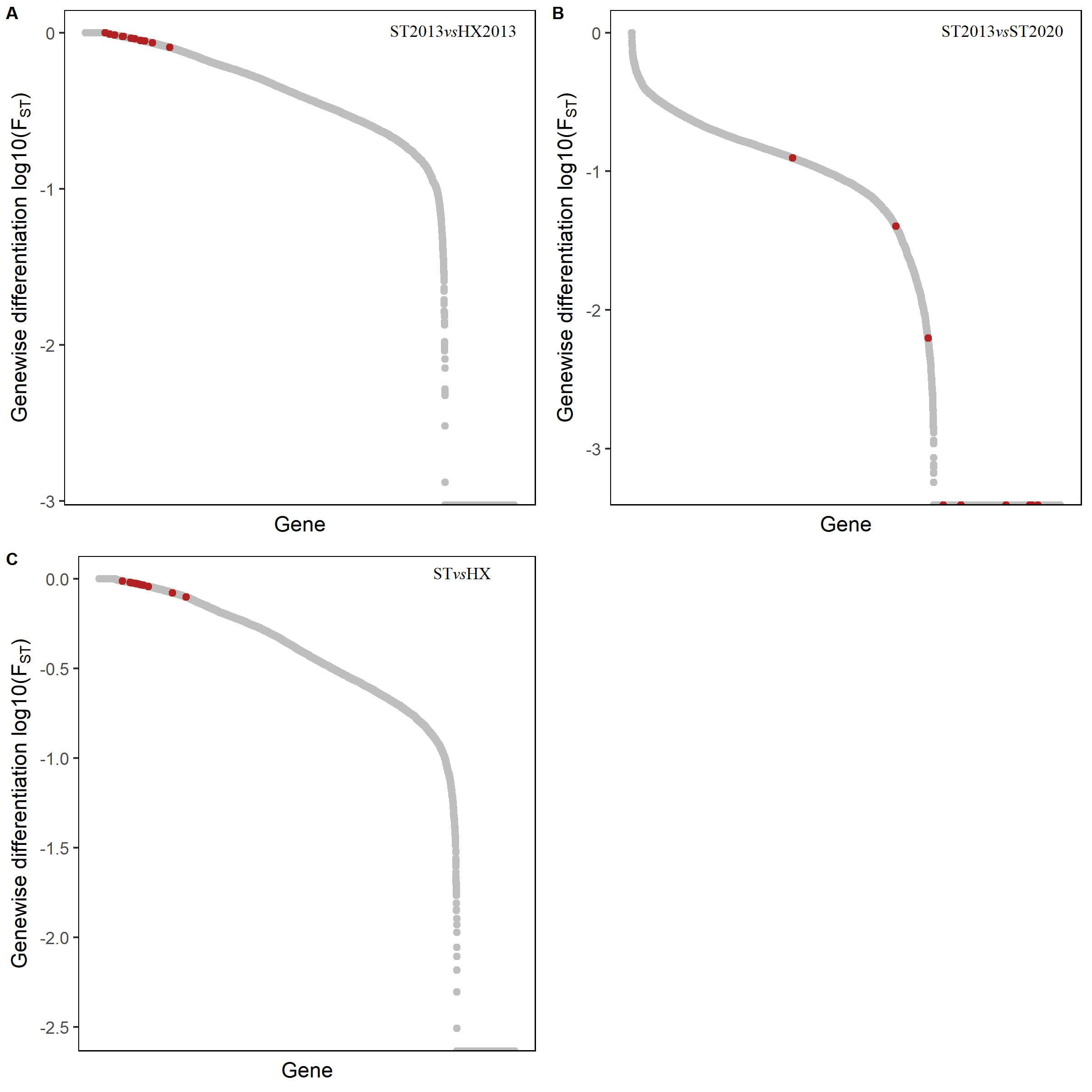

Supplement: Supplementary file 1 — Table S1: Genes of select regions in S. japonicum from ST. Table S2: Genes of select regions in S. japonicum from HX. Table S3: GO enrichment results in S. japonicum from ST. Figure S1: Box plots of (A) π ratio (πST/πHX), (B) FST, and (C) Tajima’s D of selection regions throughout genomes. Figure S2: Fisher’s exact test (A, B) and CMH test (C, D) were employed to investigate any significant SNP frequency changes (−log10[p-value]) between the two groups. −log10(P) values for common SNPs obtained through performing FET and CMH tests were plotted together (on separate axes) in order to identify significant allele frequency changes of variants in both tests (E, F). Tests between HX2013 and ST2013 are shown in (A, C, and E), while tests between ST2013 and ST2020 are shown in (B, D, and F). The red lines represent genome-wide Bonferroni’s correction of p-value. Dots in red represent SNPs of enriched genes above the genome-wide Bonferroni’s correction level. Figure S3: FST values for entire genes calculated between HX2013 and ST2013 (A), between ST2013 and ST2020 (B), and between ST and HX (C). Dots in red represent SNPs of enriched genes. [file parasite-30-59-s1.zip › Figure S3.tiff]
